# Decision rules diverge between sympatric Featherfin cichlids despite shared preferences, ecology, and evolutionary history

**DOI:** 10.1073/pnas.2620602123

**Published:** 2026-08-12

**Authors:** Maëlan Tomasek, Boyd Dunster, Zoë Goverts, Dylan Naceur, Valérie Dufour, Alex Jordan

**Affiliations:** ^a^ Laboratoire de Psychologie Sociale et Cognitive, UMR6024, CNRS, Université Clermont-Auvergne, Clermont-Ferrand 63000, France; ^b^ Behavioural Evolution Research Group, Max Planck Institute of Animal Behaviour, Konstanz 78467, Germany

**Keywords:** decision-making, perceptual load, comparative cognition, attention, decoy effect

## Abstract

It has been hypothesized that divergence in animal decision processes can be attributed to differences in information processing mechanisms. However, the required comparative tests of this idea are hindered by ecological, perceptual, or motivational divergence among species that confound a direct analysis of decision processes. To overcome these limitations, we study two closely related, sympatric cichlid fishes (*Aulonocranus dewindti* and *Cyathopharynx furcifer*) that share visual capacities, ecological context and object-removal motivations, and we perform a systematic assessment of decisional strategy divergence in wild animals, using established paradigms from human psychology research. In a series of in situ field cognitive experiments in Lake Tanganyika, comprising over 5,000 trials, we first find that the two species make similar decisions under low perceptual load, with identical size and color preferences and comparable oddity effects. Yet, when task complexity increased and choices required integrating conflicting features, the species’ decisions diverged. *C. furcifer* made rapid, consistent decisions based on a single dominant feature, whereas *A. dewindti* took longer to decide and integrated all features of the options. A decoy paradigm confirmed that *A. dewindti*’s primarily relied on available information. These findings show that closely related species can exhibit markedly different decision outcomes despite sharing sensory abilities, ecological context, and baseline preferences. The observed divergence is consistent with differences in prioritization and integration of information under complex conditions.

## Significance

When presented with the same options, can animals arrive at different decisions? Cognitive theory proposes that different species could process information differently, leading to distinct decisional outcomes. However, testing this idea is difficult because species often differ in ecology or perceptual capacities. Testing two sympatric “Featherfin” cichlids in Lake Tanganyika in a series of choice situations, we show they make identical decisions in simple contexts but diverge when decisions demand more complex information processing. One species integrates multiple features, whereas the other focuses on single cues. While this divergence could arise at different stages of the information processing pathway (e.g., upstream attentional or downstream cognitive processes), our data support a difference in attentional processes.

Understanding animal decision rules is a central challenge in cognition and evolutionary biology (1), and decision-making and information use are predicted to shape adaptive animal behavior (2). A leading hypothesis to explain variability in decisions is that species evolve different decision-making mechanisms, that is, differences in the cognitive processes (like attention, memory, or learning mechanisms) they employ when processing information (3–5). Divergence in decision-making could arise at multiple stages of the information processing pathway. Species may differ in how information is encoded and retained through learning and memory processes, or in how information is subsequently evaluated and transformed into behavioral choices. Of these, attentional processes act as the first filters of information, determining which stimuli reach downstream cognitive processes (4) and as such, small changes in attention may produce substantial differences in emergent decisions. According to load theories developed in psychology, each individual possesses a limited pool of attentional resources (i.e., the mental capacity to process information and focus on certain stimuli) that they can allocate to a situation (6, 7). In high-information situations, they must prioritize where to direct attention, and variations in these priorities can produce different outcomes from the *same* initial information. These differences in attentional resource allocation have been termed “attentional strategies,” with different strategies leading to different decisional outcomes (3). Attentional strategies thus describe the systematic ways in which animals will select, prioritize, and integrate information when making decisions. After being filtered by attentional processes, information will then be available to downstream cognitive appraisal where different species could grant unequal values to the different features of the information, favoring some over others and leading to distinct decision patterns (8). Divergence in decisions from identical initial information could thus arise at the three stages of the information processing pathway: preattentional (i.e., divergence in noncognitive processes such as sensory abilities or motivation), attentional or postattentional (i.e., divergence in cognitive evaluation).

Preattentional explanations represent the main challenge to empirical investigations of decision-making mechanisms. Indeed, many factors beyond cognition can cause species to behave differently, and studies of the cognitive mechanisms behind decision-making can consequently face numerous confounds. Factors such as perceptual scope (4, 9, 10), motivation (11), or sensory bias (12) can all influence performance in cognitive tasks, especially those conducted under natural conditions. Despite strong theoretical grounding, effective empirical tests of the role of decision-making mechanisms are therefore rare due to the many confounds that naturally arise. Ideally, a test for variation in decision-making mechanisms would use species with 1) equivalent sensory abilities, 2) equivalent motivational levels, and 3) equivalent basic preferences for all features of the options. Moreover, the capacity to perform tests with wild animals would allow their decisions to be examined in natural contexts (13–15), overcoming limitations with laboratory studies that may lack ecological validity regarding the selection pressures acting on animals (16). The Featherfin cichlids of Lake Tanganyika provide such a system, containing closely related species with shared sensory abilities, motivations, and baseline preferences that can be studied in their natural habitat.

**Fig. 1.**
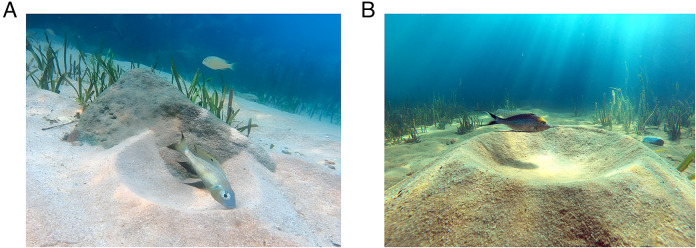
Study species. (*A*) *A. dewindti* and (*B*) *C. furcifer*.

In the present study, we use two closely related, co-occurring species of Featherfin cichlids, *Aulonocranus dewindti* and *Cyathopharynx furcifer* (Fig. 1), to study how decision-making may diverge between species, first by establishing their baseline performance and preferences in simple decision tasks, and then expanding to more complex tasks with higher perceptual demands. These two species, members of the Ectodine cichlids, are phylogenetically close, their last common ancestor being estimated at 2 Mya (17), and have equivalent visual capacities (18). They are syntopic, co-occurring in sandy bays, and form leks in which males build sand bowers to attract females (19, 20). Like bower-building birds, these species are highly motivated to maintain pristine bowers, and both immediately remove any foreign objects that fall into their bowers (20). These two species share identical decision rules when removing objects from their bower: If a shell and a stone are put inside the bower, both have robust preferences to remove the snail shell first (20, 21). However, they diverge in more complex preference reversal tasks, with *A. dewindti* showing greater inhibitory control than *C. furcifer* (20). This suggests that while baseline preferences may be shared, there is also scope for cognitive divergence in these ecologically and phylogenetically similar species.

By leveraging this balance between shared baseline preferences and divergent rules under more complex conditions, we explore whether their decision patterns also diverge and under which conditions. We first demonstrate that baseline preferences and decision patterns of the two species remain equivalent under low perceptual load. However, when we increased perceptual load by introducing conflicting stimulus features, the species diverged drastically in their decisions. These systematic differences in information use under comparable contexts suggest a divergence in how the two species prioritize and integrate information, despite sharing basic preferences.

## 1. Results

We studied adult male *A. dewindti* and *C. furcifer* on the south-eastern Zambian shore of Lake Tanganyika at depths between 3 m and 6 m. All experiments were conducted while SCUBA-diving. 3D-printed objects varying in color (blue, orange, brown, red, and green), size (1.5 cm, 2 cm, 2.5 cm, and 3 cm), and shape (*Neothauma* shell, dodecahedron, octahedron, and sphere) were used for all experiments (*SI Appendix*, Fig. S1; for the results of experiments investigating shape preferences, see *SI Appendix*, section 6).

### 1.1. Both Species Show Equivalent Preferences for Color and Size in Simple Binary Choices.

We first assessed preferences in color and size for the two species in binary choice tasks. We placed two objects varying in only one feature in the bower of the tested individual and we recorded which object was removed first (Movie S1). In total, we conducted 2,573 trials in which we tested 11 binary combinations with a minimum of six individuals per species and 90 trials per combination. Due to time and practical constraints in the field, not all combinations could be tested.

**Fig. 2.**
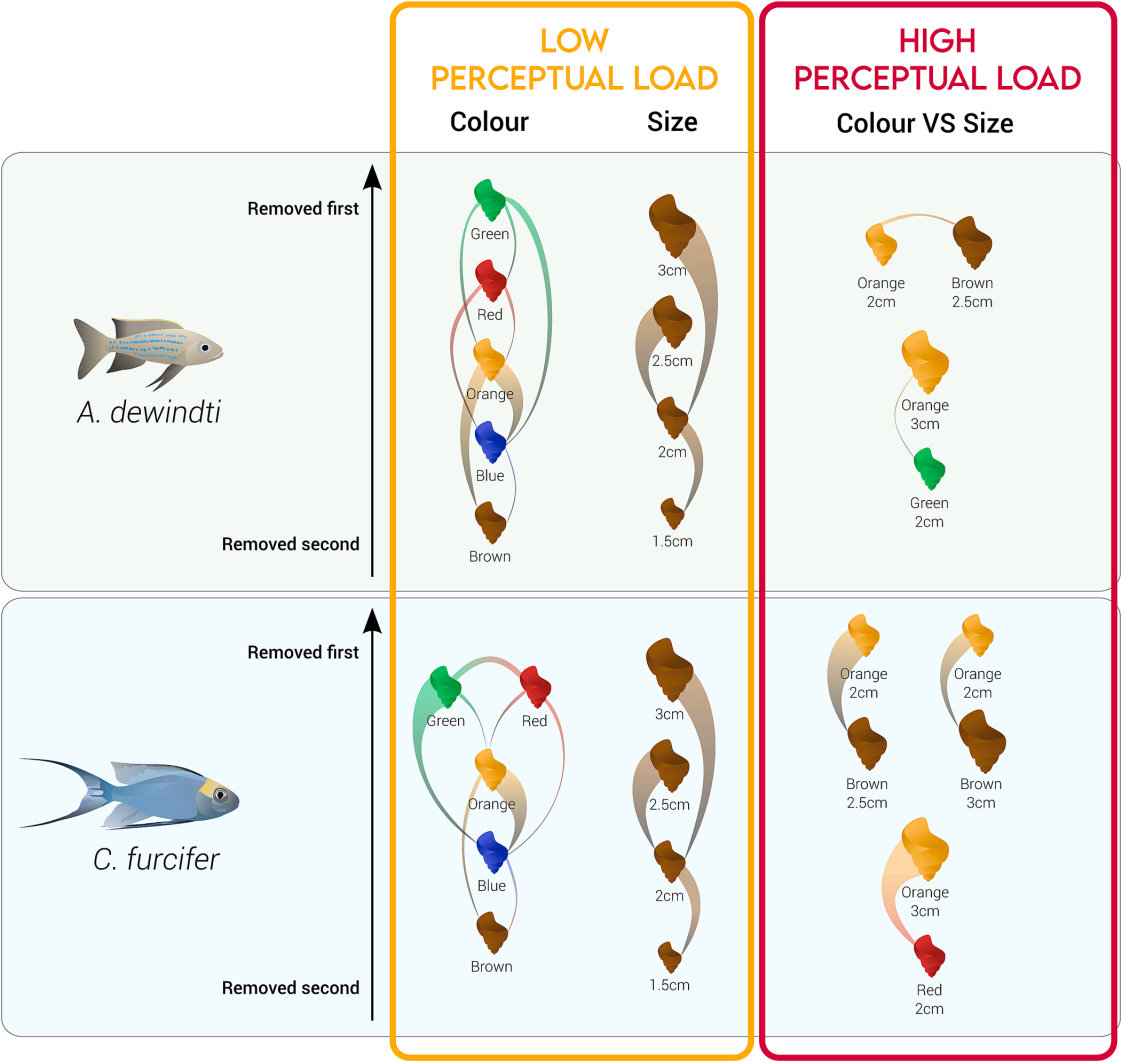
Both species show the same preference patterns in color and size (low perceptual load situation) but different decision patterns when colors and size are in conflict (high perceptual load). The relative positions of the objects are based on the number of individuals presenting a significant preference (as soon as one individual significantly preferred removing one object first over the other, this object was placed above the other) and the relative width of the links between objects indicates the proportion of individuals presenting a significant preference.

Both species presented preferences in color and size (Fig. 2 and *SI Appendix*, Figs. S2 and S3 and Table S1). First, both species presented preferences in color. The two species showed equivalent patterns regarding the direction and strength of these preferences. Only two combinations showed differences in strength: *C. furcifer*’s preferences to remove red over blue and green over blue were stronger than *A. dewindti*’s (generalized linear mixed-effect models, estimates = 0.87 ± 0.40 and 0.94 ± 0.44 respectively, *P*-values = 0.03 and 0.03 respectively, *SI Appendix*, Table S1). Second, both species preferred to remove a bigger object over a smaller one.

The two species therefore possess equivalent visual sensory abilities, motivational states, and baseline feature preferences. This equivalence in baseline preferences allowed us to eliminate noncognitive explanations for a potential decision divergence under increased perceptual load.

### 1.2. Both Species Make Equal Decisions in a Second Situation of Low Perceptual Load (Oddity Effect).

To further test whether the two species act similarly in situations of low perceptual load (situations where the animals do not have to allocate a lot of attentional resources), we offered a choice between several options which are identical except one option differing on a single feature. In this case, the decision outcome might be guided by the salient feature. This is thought to be caused by stimulus-driven or bottom–up attentional guidance, a basic attentional mechanism (3, 5, 22, 23). For instance, predators hunting a group of animals are often attracted to prey which are most noticeable among the group (24, 25). This is considered a low perceptual load situation because attention is directed toward the salient odd item, requiring minimal top–down allocation of attentional resources to distinguish the target from nontargets.

We used two colors for which neither species showed a strong preference: blue and brown. A trial consisted of the experimenter placing four 3D-printed 2 cm *Neothauma* shells in the bower simultaneously: Three shells were of the same color and one shell was of the other color (the odd shell). The trial ended once the fish had removed all shells (Movie S1). In both species, for both colors of odd shell (blue or brown), we conducted 15 trials per individual with 12 individuals, for a total of 180 trials for each color of odd shell per species (720 trials in total). If the fishes show an oddity effect, they should remove the odd shell first in more than 25% of cases.

Both species showed an oddity effect: Fishes preferred to remove the odd shell first more than other ranks of removal (*SI Appendix*, Fig. S4, ANOVA χ^2^ = 99.18, *P* < 0.001, *SI Appendix*, Table S2, see *SI Appendix*, Fig. S5 for individual results). Therefore, in situations of low perceptual load (basic preferences and oddity effect), both species make the same decisions. This in turn enables us to properly investigate their decisions in more complex choice situations.

### 1.3. The Two Species Show Different Patterns of Decision in Situations of High Perceptual Load with Conjunctions of Size and Color.

We next presented fishes with feature-conjunction choices in which objects differed in two attributes with conflicting preference rankings (e.g., preferred size but nonpreferred color vs. nonpreferred size but preferred color; Movie S1). Such situations create high perceptual load because multiple features need to be combined to evaluate trade-offs (6, 26–28). We therefore tested several size-color conjunctions, varying whether combinations presented equal or unequal trade-offs based on baseline preference strength.

Across all feature-conjunction trials (1,110 trials in 17 *A. dewindti* and 12 *C. furcifer*), the two species showed different decision patterns. *A. dewindti* consistently showed no preferences when size and color were in conflict, while *C. furcifer* showed strong preferences for one option over the other. For the first combination, *A. dewindti* showed no preference between a 2.5 cm brown shell (preferred size but nonpreferred color) and a 2 cm orange shell (preferred color but nonpreferred size). Of 16 individuals total, only one individual significantly preferred to remove the 2.5 cm brown shell first and 2 the 2 cm orange one (Fig. 2 and *SI Appendix*, Table S3). In contrast, *C. furcifer* showed a strong preference to remove the 2 cm orange shell first over the 2.5 cm brown shell (six individuals out of eight showed a significant preference, Fig. 2 and *SI Appendix*, Table S3). This difference in outcome among the two species was highly significant (generalized linear mixed-effect model, estimates = 1.54 ± 0.46, *P* < 0.001, *SI Appendix*, Table S3). Using the same individuals, we tested whether this preference was still present if the size of the shell was increased in *C. furcifer*. Although the preference vanished in some individuals, this species still showed a moderate preference to remove the 2 cm orange shell first over the 3 cm brown shell (three individuals out of eight still showed a significant preference, Fig. 2 and *SI Appendix*, Fig. S6 and Table S3).

To ensure this pattern was not specific to one set of colors, we tested a second combination using different colors with equivalent baseline preference strengths in both species (*Materials and Methods*). *A. dewindti* again showed no preference to remove a 3 cm orange shell (preferred size but nonpreferred color) over a 2 cm green shell (preferred color but nonpreferred size; 3 individuals out of 16 showed a significant preference). In contrast, *C. furcifer* showed a strong preference to remove a 3 cm orange shell (preferred size but nonpreferred color) over a 2 cm red shell (preferred color but nonpreferred size; six individuals out of eight showed a significant preference to remove the larger orange shell first, and one individual showed a significant preference to remove the smaller red shell first, *SI Appendix*, Fig. S6 and Table S3). When this latter individual was removed from the analysis, there was a significant difference in the decision patterns of the two species (generalized linear mixed-effect model, estimates = −1.06 ± 0.34, *P* < 0.001, *SI Appendix*, Table S3).

**Fig. 3.**
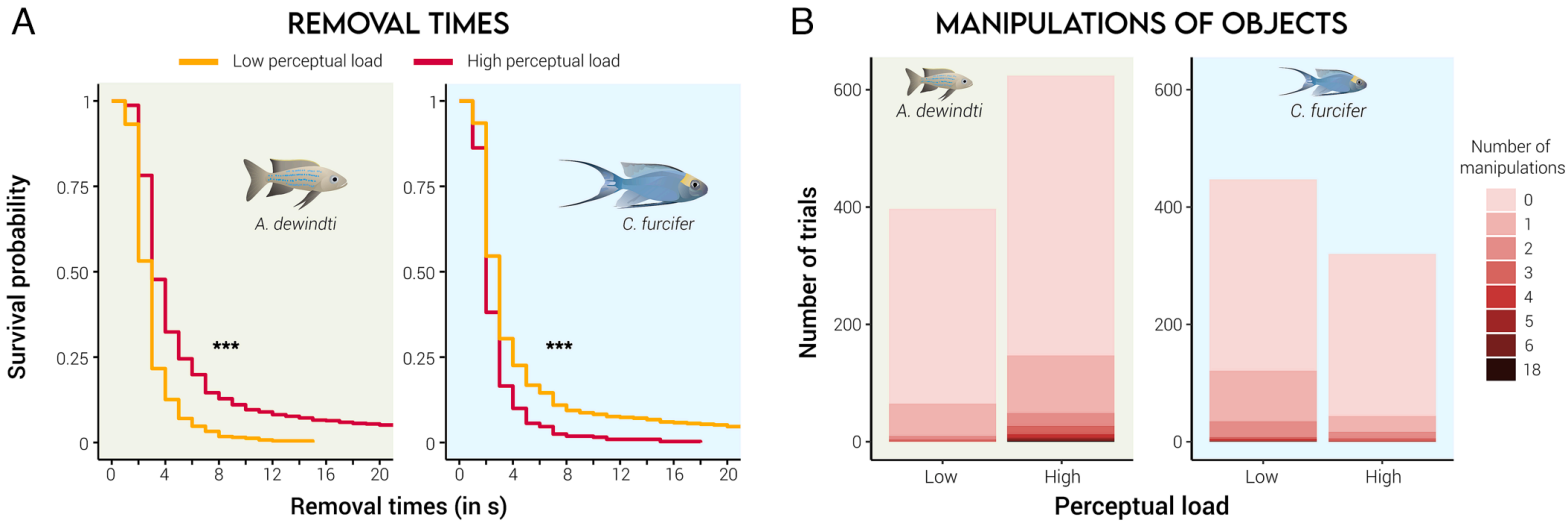
*A. dewindti* takes longer to decide in situations of high perceptual load while *C furcifer* is quicker. (*A*) Latency to remove the first object depending on the perceptual load (***P < 0.001, least-square means post hoc tests after Cox regression models). (*B*) Manipulations of the objects inside the bower before making a decision (no significance in the two species between the two levels of perceptual load).

Decision latencies and object manipulations provided additional evidence for divergent information processing. *A. dewindti* took significantly longer to remove objects in high-load (conjunction) vs. low-load (binary) trials (least-square means post hoc test after Cox regression model, estimates = −0.50 ± 0.08, *P* < 0.001, Fig. 3*A* and *SI Appendix*, Table S4). In contrast, and against expectation, *C. furcifer* took less time to remove objects in high-load trials than in low-load trials (estimate = 0.43 ± 0.09, *P* < 0.001; Fig. 3*A*). Species showed similar latencies to remove objects in situations of low perceptual load (least-square means post hoc test after Cox regression model, estimates = 0.16 ± 0.14, *P* = 0.68, Fig. 3*B* and *SI Appendix*, Table S5), confirming that baseline motivation and engagement were equivalent. Neither species showed significantly increased object manipulation in high-load trials (Fig. 3*B* and *SI Appendix*, Table S5).

Together, these results demonstrate that despite equivalent performance in low-load situations, the two species employ different decision-making strategies when perceptual load increases. *C. furcifer* makes rapid choices even when features conflict, while *A. dewindti* shows prolonged decision times and no clear preference. This divergence could reflect differences in attentional capacity, attentional prioritization or downstream evaluation of information. The lack of preference in *A. dewindti* could be explained by a complete dismissal of the different features presented by the options, and therefore random choices. On the other hand, as suggested by their longer decision times, *A. dewindti* may process all of the information contained in the options and consider them equal. We conducted an additional experiment with this species to disentangle these possibilities.

### 1.4. *A. Dewindti* Considers All Features of the Objects in the Feature-Conjunction Situation (Decoy Effect).

Here we investigated how *A. dewindti* behaves in a three-option situation by using a decoy effect paradigm. Such tasks consist of adding a third inferior option (the “decoy” option), that can shift preferences between two initially equivalent options (8). In a choice among options A and B, which are equally preferred, the introduction of a third option C (the decoy), which is less preferred than B (the “competitor” option) in one feature and less preferred than A (the “target” option) in two features, can increase preference for the target relative to the competitor. If *A. dewindti* chose randomly in the previous feature conjunction situations, it should also choose randomly among all three options here (33% each for A, B, and C). However, if *A. dewindti* did integrate both features but found the options genuinely equivalent, it should be immune to the decoy effect, continuing to show no preference between target and competitor while avoiding the inferior decoy.

We used decoys for size and for color (Movie S1). In the size decoy experiment, we used a 2 cm orange shell (target option) and a 2.5 cm brown shell (competitor option) for which the fishes showed no preference in the feature-conjunction experiment. We added a 1.5 cm brown shell as a third option (decoy option) which, given the results of the basic preferences, was less preferred than the competitor in one feature (size) but less preferred than the target in both features (size and color). In the color decoy experiment, the decoy option was a 2 cm blue shell which was added to a 3 cm orange shell (target option) and a 2 cm green shell (competitor option). This third option was less preferred than the competitor in one feature (color) but less preferred than the target option in both features (color and size). We could not conduct these experiments with *C. furcifer* because this species already showed strong preferences in the conjunction experiment, violating the prerequisite of initial indifference for the options, and this test aimed at disentangling whether *A. dewindti* was choosing randomly or not in the feature-conjunction situation, which *C. furcifer* demonstrably was not. We only tested *A. dewindti* individuals which did not show an initial significant preference for the target options during the feature-conjunction experiment (n = 14 for size decoy, n = 12 for color decoy; minimum 20 trials per individual; 599 total trials).

**Fig. 4.**
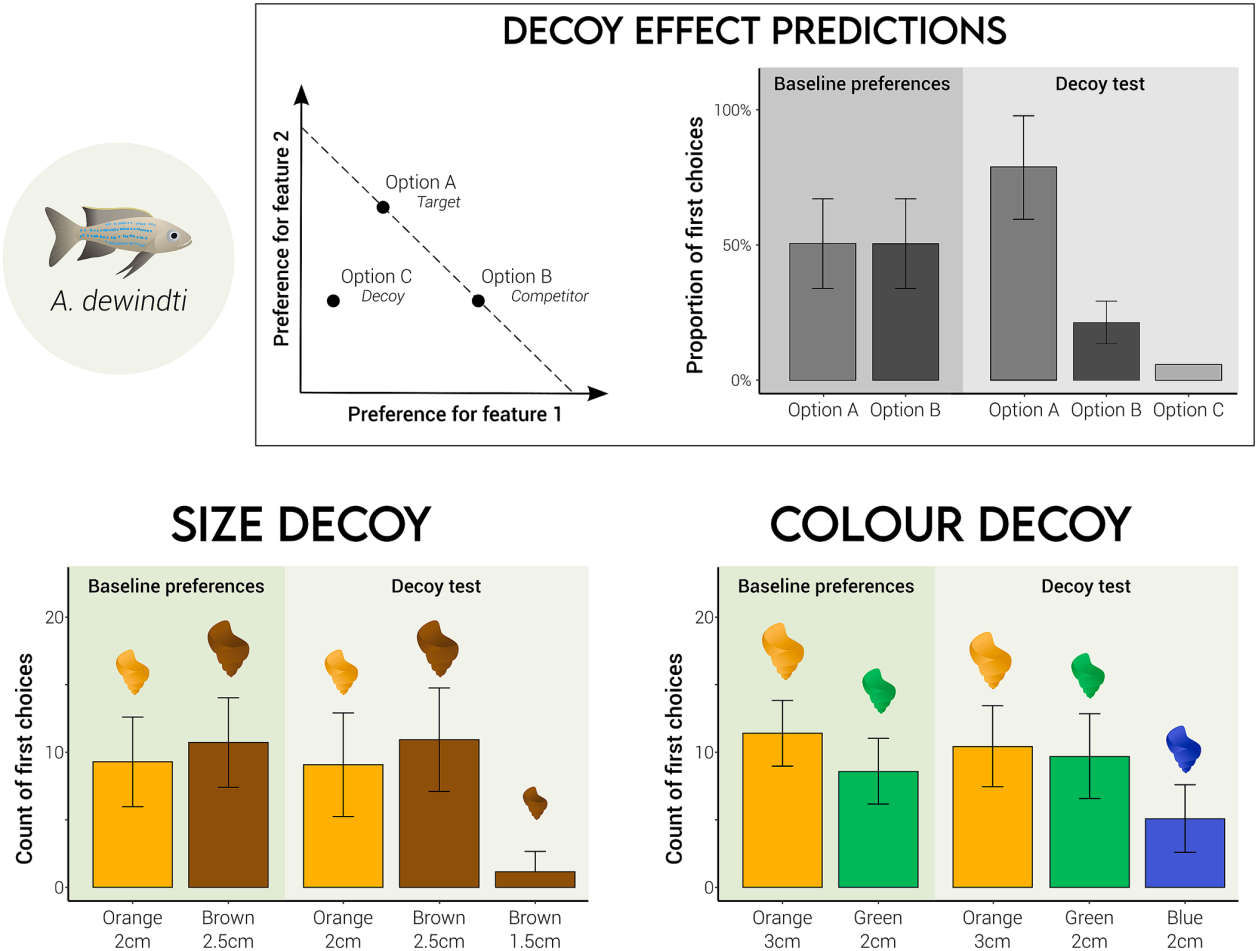
*A. dewindti* shows no evidence for a decoy effect. In this situation, Option C (decoy) is less preferred than option B (competitor) on one feature only but less preferred than option A (target) on two features. If fishes present a decoy effect, their initial relative preference for options A and B should change (see predictions in the *Upper* panel). We conducted both a size decoy test (n = 14 individuals) and a color decoy test (n = 12 individuals). *Left* panels indicate the baseline preference between the target and competitor options in binary choice tasks and *Right* panels show the results of the decoy tests. If *A. dewindti* was sensitive to a decoy effect, the ratio of preferences between target and competitor options should have been modified with the addition of the decoy option. These results confirm that this species considers all available information in a situation of high perceptual load.

**Fig. 5.**
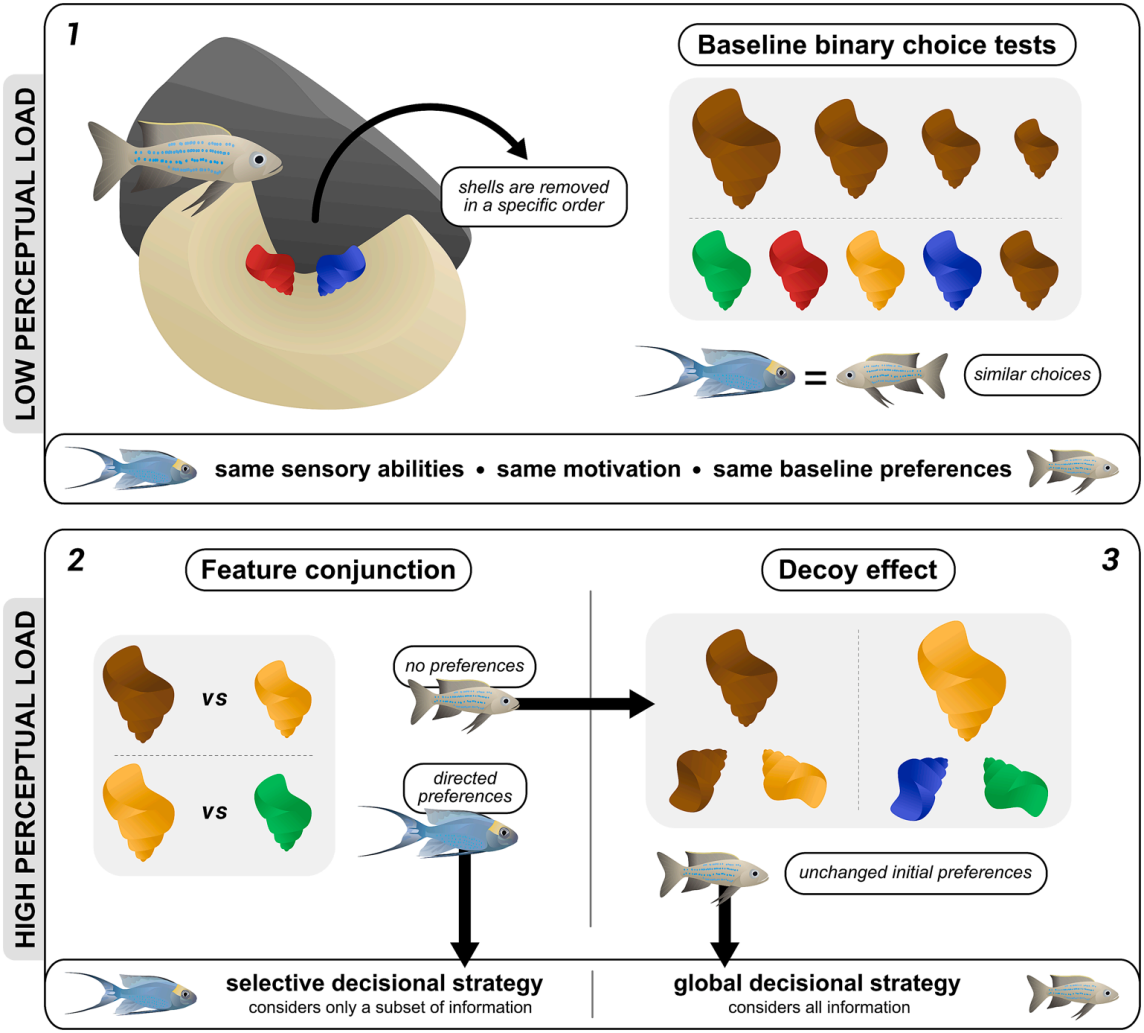
Graphical abstract summarizing major results.

*A. dewindti* did not decide randomly nor did it show sensitivity to decoy effects in either experiment (Fig. 4). In the size decoy experiment, individuals maintained their baseline 50:50 preferences between target and competitor options; only 3 of 14 individuals showed significant shifts in relative preference (2 toward target, 1 toward competitor; *SI Appendix*, Table S6*A*). In the color decoy experiment, no individual switched its relative preferences from baseline preferences to the decoy test (*SI Appendix*, Table S6*B*). Overall, *A. dewindti*’s decisions when size and color were in conflict were not sensitive to a decoy effect, suggesting that individuals were able to consider multiple stimulus dimensions simultaneously rather than being driven by a single cue (Fig. 5).

## 2. Discussion

We demonstrate that two closely related cichlid species that have equal visual capacities and motivational levels, *A. dewindti* and *C. furcifer*, make identical decisions under low perceptual load but diverge under high perceptual load. In natural experiments, we showed that the two species showed similar color and size preferences in binary choices and displayed equivalent oddity effects, confirming that baseline sensory abilities, motivations, and stimulus-driven attention are matched. This equivalence in performance allowed us to investigate decision-making mechanisms in the two species and compare them on equal terms. By increasing the perceptual load of the choice scenarios using sets of feature conjunctions, we found diverging decision patterns as the tasks increased in complexity. *C. furcifer* decided quickly and showed a strong preference to remove one of the two objects first, apparently based on a single dominant feature. In contrast, *A. dewindti* showed longer decision times and balanced consideration of multiple features and was also unaffected by a decoy paradigm for size or color (8, 29), suggesting this species integrates and processes all available information in feature-conjunction situations. Taken together, our results reveal a fundamental aspect of cognitive biology, in which subtle changes in information processing can lead to divergent decision outcomes.

Divergence in decisions between species may be explained at three points along the information processing pathway: preattentional, attentional, or postattentional. First, preattentional mechanisms may explain divergence in decisions, such as different sensory abilities, motivation, or innate attraction to certain features. Because sensory capacities, motivation, and basic preferences are equivalent in the two species under consideration, the first pathway is unlikely. Noncognitive factors, like a different distribution of personality profiles or emotional states, if they exist, could explain behavioral differences in high perceptual load situations, but the similar behaviors of the two species in low-load situations renders these possibilities unlikely. The explanation for the divergence is thus more likely to be found downstream of the information processing pathway. Two alternative attentional explanations are potentially consistent with the observed divergence in decisions. First, *C. furcifer* may possess relatively lower attentional resources than *A. dewindti,* limiting their access to information about the options in situations of higher perceptual load (30, 31). Alternatively, the two species may possess equal attentional capacities but use different prioritization strategies. *C. furcifer* may indeed weigh some features differently under high perceptual load, displaying different attentional priorities and leading to divergent decisions in the two species (3). Our current data cannot definitively distinguish between these alternatives, though several lines of evidence support a difference in attentional strategies rather than capacities. First, *C. furcifer* responded faster in the more difficult task, which appears to be inconsistent with capacity overload in which response times typically increase. Second, *C. furcifer*’s strong, consistent preferences suggest at least some consideration of the different features of the options, contrary to more random choice patterns should their attentional resources be overwhelmed. Third, and in keeping with Morgan’s canon, the rapid evolutionary timescale (~2 My) suggests that the existing cognitive machinery is being employed differently in these species rather than one or both having evolved greater or more reduced attentional capacities. Further studies are needed to pinpoint potential differences in attentional abilities among the species, evaluating for instance attentional capture by distractors in situations of varying perceptual loads (6) or attentional prioritization of multiple features in other cognitive processes, like categorization (32). Finally, the divergence in decisions in high perceptual load situations could be explained by postattentional differences. In that case, the two species would cognitively evaluate the shape and size of objects differently. For instance, some species of butterflies prioritize color over scent while visiting flowers (33). However, given the inconsistent prioritization of features in *C. furcifer* and the higher cognitive demand this explanation requires, it is inconsistent with Morgan’s canon, and we believe attentional explanations to be more parsimonious.

If differential expressions of attentional strategies explain the divergence in decisions in high perceptual load situations, we can postulate about the origins of this divergence. Experiments with humans show that attentional strategies can be very plastic and that environmental differences can produce qualitatively distinct attentional profiles. For instance, populations living in a visually sparse rural environment display a stronger local bias than populations living in more visually complex urban environments, who tend toward a more global processing of visual scenes, but this local bias can be modified by a simple exposure to urban environment (34–36). Similarly, past experience can affect attentional strategies, for instance by making individuals focus on features that have had rewarding significance in the past (37–39). Socio-ecological and life history traits could therefore explain the propensity of the individuals of a species to use one attentional strategy over another. Our two species are similar in their social and life history traits but diverge in two ecological traits: *C. furcifer* feeds on algae while *A. dewindti* feeds on plankton; and *C. furcifer* builds its bowers exclusively on flat sand or rock surfaces, while *A. dewindti* builds them on small sand patches among complex rock faces (20, 40). This ecological complexity in the latter species could shape more enhanced multiattribute decision-making and the ability to maintain multiple feature representations when assessing potential bower sites. In contrast, *C. furcifer*’s less complex, flat-substrate habitats may lead to reliance on simpler decision rules with individuals favoring rapid, decisive choices based on a single dominant feature rather than prolonged multiattribute integration.

Our study provides rare empirical evidence of the dynamics of decision-making between closely related sympatric species in their natural habitat. By comparing two cichlid fishes with minimal confounding differences, we demonstrate that pronounced behavioral divergence can emerge from subtle modifications in the distribution of decision-making strategies. By using perceptual load paradigms originally developed in humans, in which identical stimuli are processed differently depending on whether a participant focuses on an isolated feature or processes the conjunction between several features, we show that our two species display identical low-level decisions but adopt different selectivity patterns under high perceptual load. Our findings highlight attentional strategies as a major potential area of interest to explain decision divergence in animals and answer a call for a much-needed interdisciplinary cooperation between animal cognition, psychology, evolutionary biology, and ecology, which will hopefully be pursued in the future (32).

## 3. Materials and Methods

Our study demonstrates the feasibility of cognitive testing in wild populations under natural conditions. The bower-maintenance behavior of these cichlids provides a naturally occurring, ecologically relevant context for testing complex cognitive processes without the confounds introduced by captivity (16, 41). Critically, the system allows control of typically problematic confounds, namely differences in sensory abilities, motivation, and baseline preferences, that have hindered previous comparative cognitive studies (42). The approach may be particularly valuable for testing cognitive evolution in other species with stereotyped, stimulus-triggered behaviors that can be exploited for experimental manipulation in nature.

### 3.1. Subjects.

Adult male *A. dewindti* and *C. furcifer* were found along the south-eastern Zambian shore of Lake Tanganyika (Isanga Bay, 8°37′24.7″S, 31°12′02.9″E) at depths between 3 m and 6 m. Of about 25 individuals of each species tested for candidacy, 18 *A. dewindti* and 16 *C. furcifer* were responsive and tested in at least one experiment. Responsive individuals were defined as such when they removed items from their bower within a maximum of 3 min. Note that they usually did so in a matter of seconds. Individuals could be recognized from day to day because bower-holding males hold the same bower for an extended period of time (43). They could also be further distinguished based on physical differences (size, body markings, and unvarying color hues). Experiments were conducted while scuba diving and took place between 06:00 and 11:00 AM between April 6th and May 13th, 2024. An external clinical examination of all potential candidates was conducted by a qualified veterinarian (MT) and revealed no obvious health issue.

### 3.2. Binary Choice Tasks.

Binary choice tasks were used to assess the preference of the fishes to remove one object first when presented with two objects in their bower. A trial consisted of the experimenter placing two 3D-printed objects (polylactic acid) inside the bower of a fish and waiting until both were removed. Objects were placed in the center of the bower, a few centimeters apart, in a standardized position. To avoid location biases, their relative positions were alternated at each trial. A session consisted of five consecutive trials. If several sessions were conducted with the same individual within the same dive, the experimenter swam away for at least 3 min between sessions. For each binary combination, the minimal experimental structure consisted of three sessions of five trials in six individuals per species, for a total of 90 trials per species. Departures from this basic structure occurred 14 times out of 39 experiments in total, either when more than six individuals were tested (to obtain baseline preferences for further experiments or because some individuals became unreactive during the experiment) or when more trials were conducted per individual.

To assess basic preferences, objects varied in only one attribute: color, size, or shape. For color, we used 3D-printed *Neothauma* shells of 2 cm length varying in color (blue, orange, brown, red, and green). For size, we used 3D-printed *Neothauma* brown shells varying in length (1.5 cm, 2 cm, 2.5 cm, and 3 cm). For shape, we used 3D-printed brown objects of either 2 cm or 3 cm length varying in shape (*Neothauma* shell, dodecahedron, octahedron, and sphere, *SI Appendix*, section 6). Note that due to time and practical constraints in the field (not all objects could be fabricated and no 3D-printer was accessible), not all combinations could be tested. In situations of high perceptual load, each object was defined by a conjunction of features in conflict with each other (e.g., one object with a preferred size but nonpreferred color and the other object with nonpreferred size but preferred color).

Each session was video recorded and we scored the first object removed from the bower in each trial (defined as the first object crossing the rim of the bower). If an individual clearly showed an intent of removing an object first (grabbing and displacing it) but let this one slip from its mouth before it crossed the rim of the bower (which happened in 2% of all trials, 84 times out of 4,792 trials), we counted this attempt as the first object removed.

### 3.3. Oddity Effect.

We investigated how the two species reacted in a second situation of low perceptual load to test whether their decisions would be guided by a salient feature. More precisely, we presented a set of three equal options and one odd option. We used two colors for which most individuals in the two species had not shown a preference: blue and brown (only 4 out of the 24 tested individuals had shown a significant preference for one of the two colors in baseline preference assessments, *SI Appendix*, section 2). A trial consisted of the experimenter placing four 3D-printed 2 cm *Neothauma* shells in the bower simultaneously: Three shells were of the same color, and one shell was of the other color (the odd shell). The trial ended once the fish had removed all shells. The shells were placed simultaneously in the bower in a standardized way with the tips facing each other, and the position of the odd shell was alternated at each trial. In the two species, for both colors of odd shell (blue or brown), we conducted three sessions of five trials per individual with 12 individuals, for a total of 180 trials for each color of odd shell per species. Each session was video recorded, and we scored the rank of removal of the odd shell (whether removed first, second, third, or last) for each trial.

### 3.4. Decoy Effect.

All initial ratios of preferences were previously assessed during the feature conjunction experiment. We only kept *A. dewindti* individuals which did not present an initial significant preference for the target options. Thus, in total, we investigated the decoy effect of size in 14 individuals and of color in 12 individuals (*SI Appendix*, section 2). A trial consisted of the experimenter placing the three options in the bower and waiting for the fish to remove all shells. All shells were placed in a standardized way with the tips facing each other, and the positions of the shells were alternated at each trial. A session consisted of five trials. To enable a thorough comparison with the baseline preference experiments which consisted of 20 trials per individual, we conducted as many sessions of decoy tests as necessary to obtain 20 trials with either the target or the competitor option removed first. In a session of five trials, if the fish removed the decoy option first less than twice, the session was prolonged to have a total of five trials in which the fish removed either the target or the competitor option first (the maximum number of trials attained within such a session was nine). In a session of five trials, if the fish removed the decoy option first more than twice, the session was ended and another session was conducted later on to complete the missed trials (the maximum number of sessions that an individual did was five). Each session was video recorded and we scored the removals of each option.

### 3.5. Quantification and Statistical Analysis.

All statistical analyses were done using R (version 4.2.2).

#### 3.5.1. Binary choice tasks.

In each species and for each combination of objects, we ran a generalized linear mixed-effect model [Object removed first ~ Subject + (1|Session/Trial)] with a binomial family distribution [package “glmmTMB” in R (44)]. For each generalized linear model, we analyzed the residuals to explore the fit of the models [“DHARMa” package in R (45)]. We thus obtained preferences for removing one object first at the individual level.

To investigate species differences in the decision patterns (direction and/or strength), for each combination, we ran generalized linear mixed effect models [Object removed first ~ Species + (1|Subject) + (1|Session/Trial)] with a binomial family distribution. Residuals were analyzed as described above.

We scored the removal latencies (defined as the time between the individual “seeing” the objects in the bower—either when the fish returned and crossed the rim of the bower or when the fish clearly switched direction to swim toward the bower, while being at a height enabling him to see inside—and the removal of the first object) and the number of manipulations of objects (touching or nudging the objects before removing the first one) in both situations of low and high perceptual load. For situations of low perceptual load, we scored them in four combinations for each species: 2 cm orange shell vs. 2 cm brown shell, 2 cm brown shell vs. 2.5 cm brown shell, 2 cm brown shell vs. 3 cm brown shell, 2 cm orange shell vs. 2 cm green shell for *A. dewindti* and 2 cm orange shell vs. 2 cm red shell for *C. furcifer*. For situations of high perceptual load, we scored them in two combinations per species: 2 cm orange shell vs. 2.5 cm brown shell, 2 cm green shell vs. 3 cm orange shell for *A. dewindti*, 2 cm red shell vs. 3 cm orange shell for *C. furcifer*. To investigate the removal latencies, we conducted a Cox regression model [Removal latency ~ Perceptual load * Species + (1|Subject)] [package “coxme” in R (46)]. We checked for proportional hazards with a Schoenfeld test [function “cox.zph,” package “survival” in R (46)]. We then ran least-square means post hoc tests to investigate decision times within species and perceptual loads [function “lsmeans,” lsmeans package in R (47). To investigate the number of manipulations of objects before making a decision, we ran a generalized linear mixed-effect model with a negative binomial family (Number of manipulations ~ Perceptual load * Species + (1|Subject)]. We then ran least-square means post hoc tests as described above.

#### 3.5.2. Oddity effect.

To investigate an oddity effect at the population level, we ran a generalized linear mixed effect model [Total of removals ~ Rank * Species * Color of the odd shell + (1|Subject)] with a Poisson family distribution. The variable “Rank” is the position in which the odd shell was removed (whether first, second, third, or last shell removed). Residuals of the model were analyzed as described above. We then ran a type II ANOVA to analyze the contribution of each factor and their interactions [package “car” in R (48)].

To investigate an oddity effect at the individual level, for each subject, we conducted Pearson’s chi-square tests [package “stats” in R (49)] to analyze whether the distribution of the ranks of removal of the odd shell was significantly different from a uniform distribution.

#### 3.5.3. Decoy effect.

To investigate whether the ratios of preferences changed between the baseline preference experimental condition or the decoy effect experimental condition, we ran a generalized linear mixed effect model [Object removed first ~ Experimental condition * Subject + (1|Sessionfi/Trial)] with a binomial family distribution. An analysis of the residuals and a type II ANOVA were conducted as described above. We then ran least-square means post hoc tests to analyze whether specific individuals had modified their initial relative preference in the presence of the decoy.

### 3.6. Ethical Approval.

We declare that our study is in full accordance with the ethical guidelines of our institutions and comply with the Zambian and European legislation on animal welfare. The experiments were conducted according to the Guidelines for the treatment of animals in behavioural research and teaching (https://doi.org/10.1016/S0003-3472(21)00389-4) and were conducted under Zambian Study Permit numbers C-24173000/4-24 and SP297185/4-22.

## Supplementary Material

Appendix 01 (PDF)

Dataset S01 (XLSX)

Code S01 (TXT)

Movie S1. **Videos of the series of experiments**. (binary choice task in low perceptual load, test of oddity effect, binary choice task in high perceptual load, test of decoy effect)

## Data Availability

Study data are included in the article and/or supporting information.
