# Supplementary material for: Decision rules diverge between sympatric Featherfin cichlids despite shared preferences, ecology, and evolutionary history
Source: Proc Natl Acad Sci U S A. 2026 Aug 12;123(33):e2620602123. doi: 10.1073/pnas.2620602123 (PMC13486600; doi:10.1073/pnas.2620602123)
Supplement: Supplementary file 1 [file pnas.2620602123.sapp.pdf]

Supplementary materials

SECTION 1: Experimental material

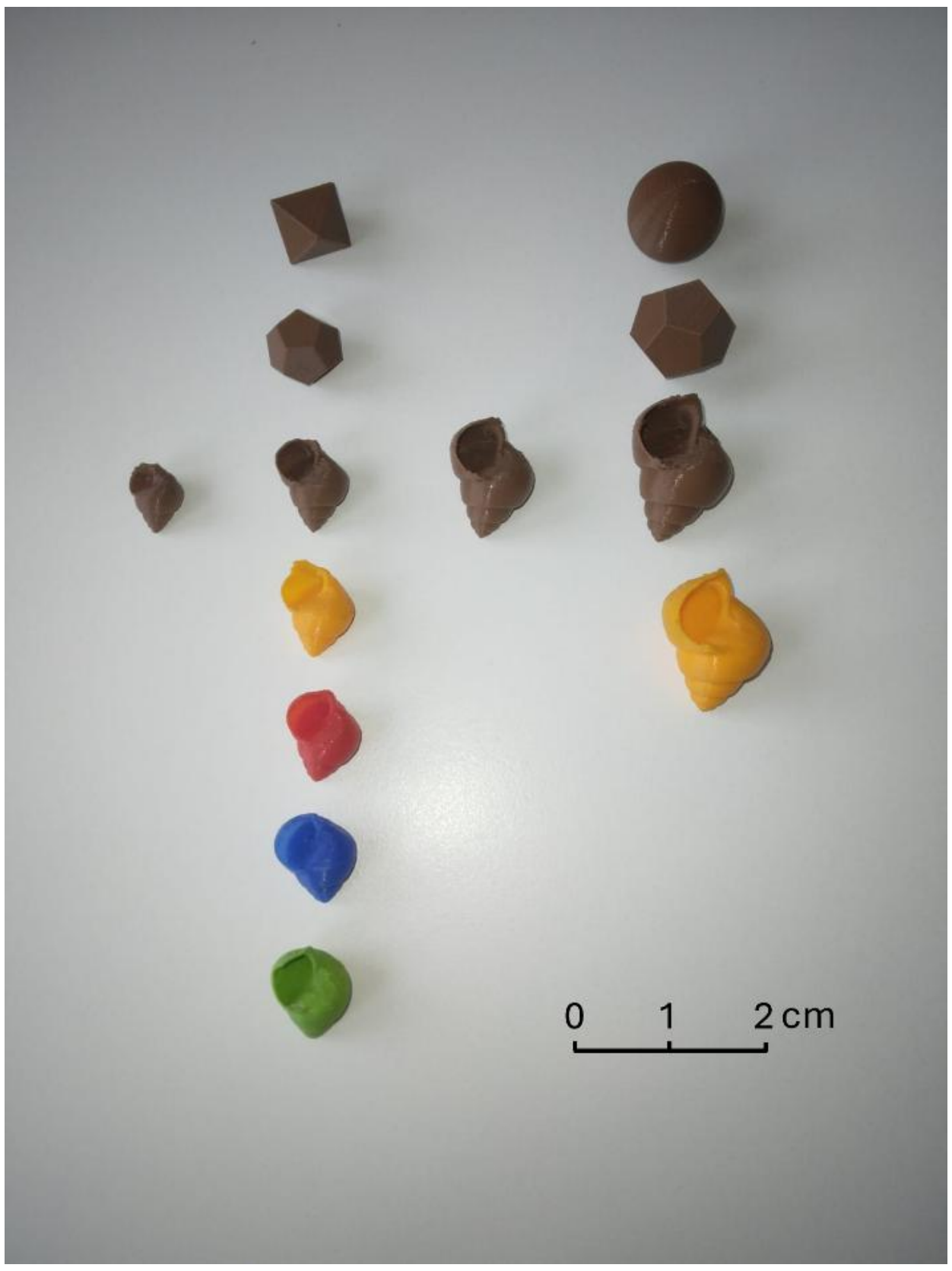

Figure S1: 3D-printed objects used in the various experiments.

SECTION 2: Basic preferences, binary choices

Table S1. Results of the basic preferences. Are indicated the number of total first removals in the whole population for each binary choice, the number of individuals presenting a significant preference for removing one option first or no preference (GLMM Object removed first ~ Subject + (1|Session/Trial), binomial distribution), and the results of the interspecific differences in decision patterns (GLMM Object removed first ~ Species + (1|Subject) + (1|Session/Trial), binomial distribution).

**BASIC PREFERENCES** (binary choices)										
**Colour** (Shells, 2cm)										
Colour A	Colour B	Species	Number of choices		Number of individuals			Interspecific difference		
			A	B	Preference for A	No preference	Preference for B	Estimate	Standard error	p-value
Blue	Brown	Dewindti	92	98	1	10	0	-0.23	0.37	0,54
		Furcifer	107	90	3	9	1			
Blue	Red	Dewindti	35	65	0	6	1	0.87	0.40	**0,03 ***
		Furcifer	20	70	0	5	1			
Blue	Green	Dewindti	25	65	0	4	2	0.94	0.44	**0,03 ***
		Furcifer	12	79	0	0	6			
Blue	Orange	Dewindti	36	144	0	2	4	-0.29	0.52	0,59
		Furcifer	41	136	0	1	5			
Brown	Orange	Dewindti	26	64	0	3	3	-0.29	0.50	0,56
		Furcifer	59	31	0	5	1			
Green	Orange	Dewindti	59	32	1	5	0	0.38	0.39	0,34
		Furcifer	50	40	1	5	0			
Orange	Red	Dewindti	41	49	1	5	0	0.38	0.38	0,32
		Furcifer	33	57	0	5	1			
Green	Red	Dewindti	49	41	1	5	0	0.07	0.67	0,92
		Furcifer	48	42	2	2	2			
**Size** (Shells, brown)										
Size A	Size B	Species	Number of choices		Number of individuals			Interspecific difference		
			A	B	Preference for A	No preference	Preference for B	Estimate	Standard error	p-value
1.5cm	2cm	Dewindti	18	73	0	3	3	-0.39	0.42	0.35
		Furcifer	24	66	0	3	3			
2cm	2.5cm	Dewindti	38	143	0	0	6			
		Furcifer	52	143	0	1	6			
2cm	3cm	Dewindti	7	83	0	0	6	-0.81	0.50	0.11
		Furcifer	14	76	0	1	5			

Figure S2: Results of the binary choices in basic preferences binary choices for *A. dewindti*: Models: Choice ~ Subject + (1|Session/Trial), Binomial family

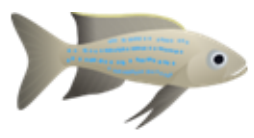

A. dewindti

Color

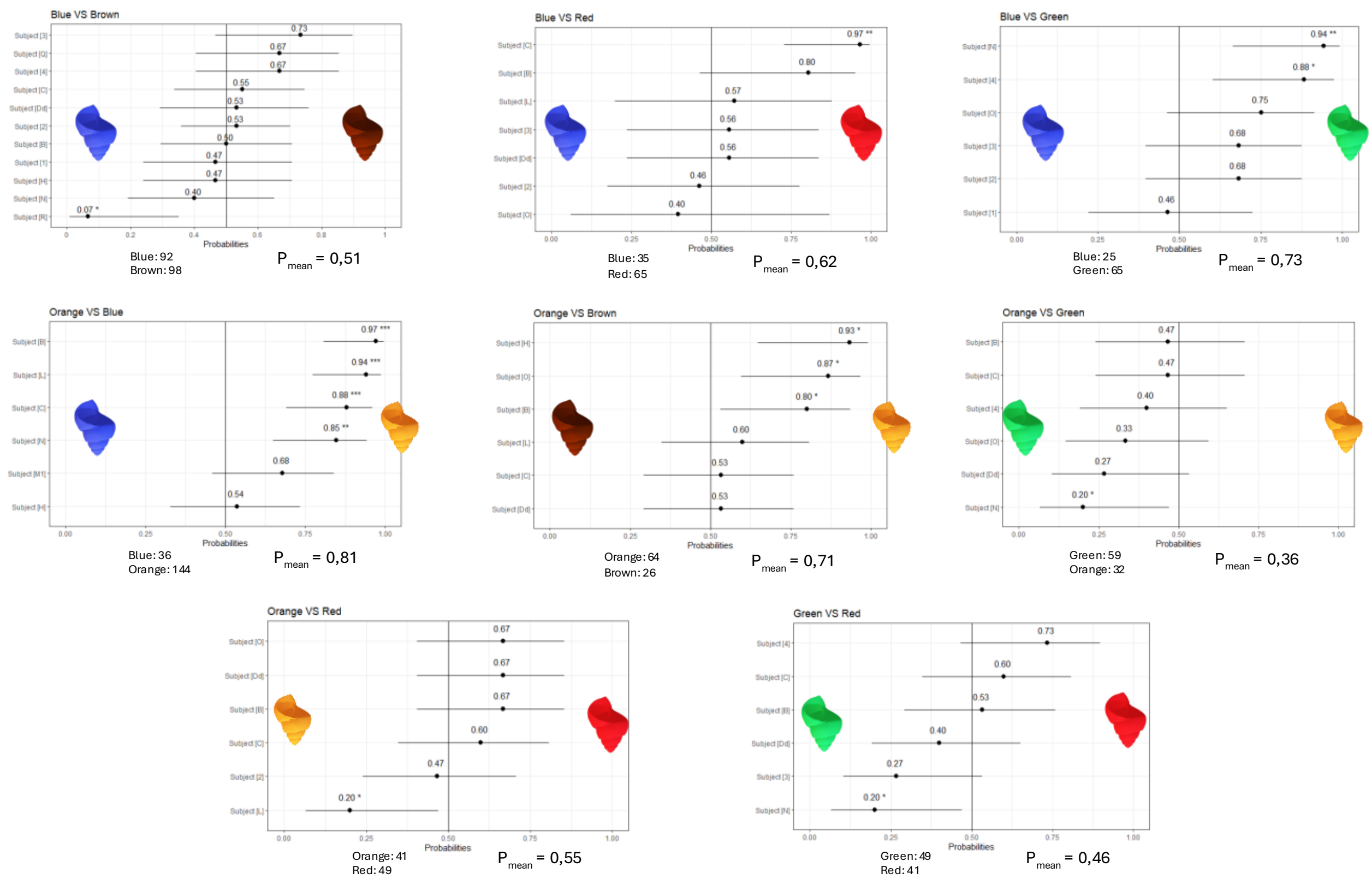

Size

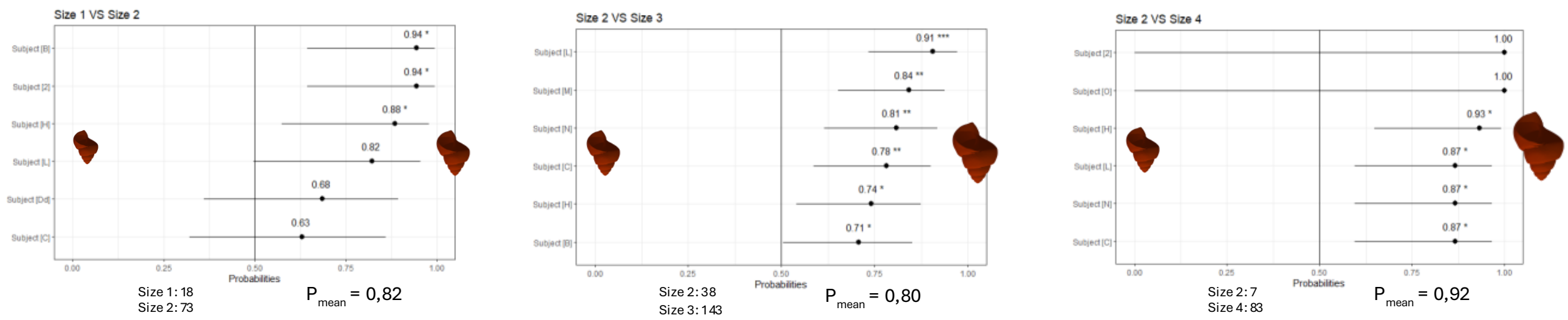

Figure S3: Results of the binary choices in basic preferences binary choices for *C. furcifer*:

Models: Choice ~ Subject + (1|Session/Trial), Binomial family

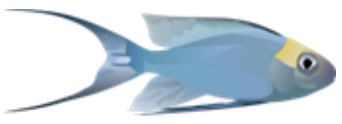

C. furcifer

Color

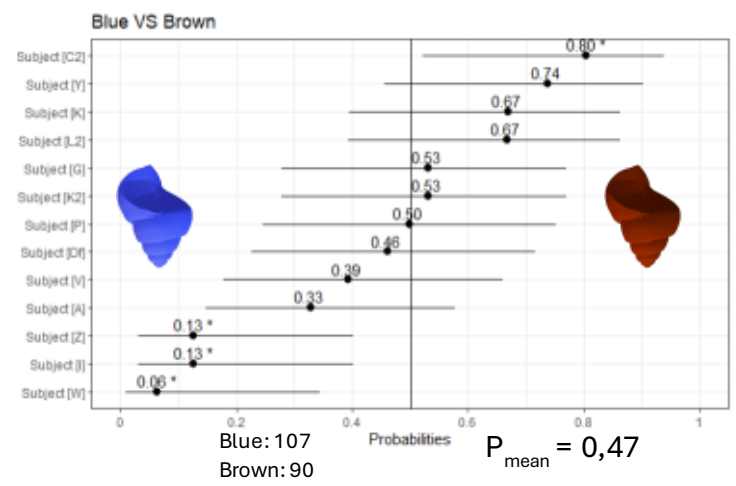

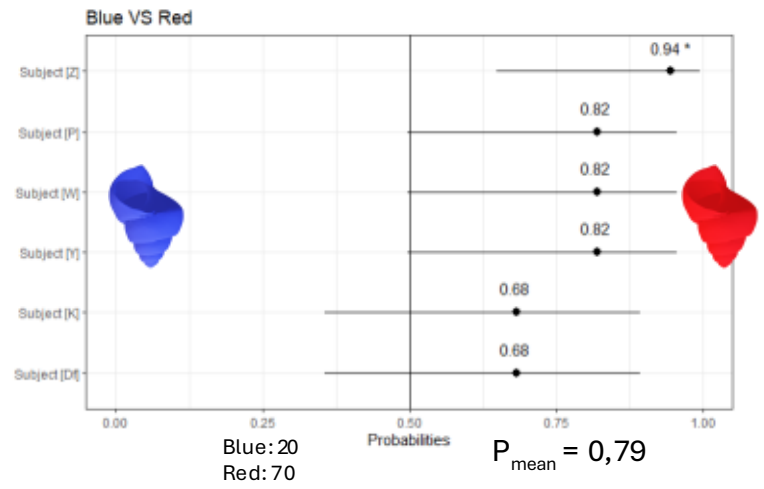

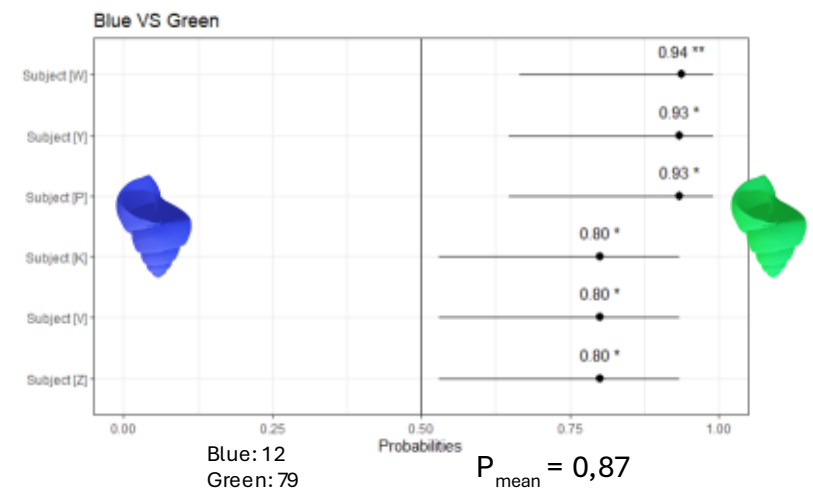

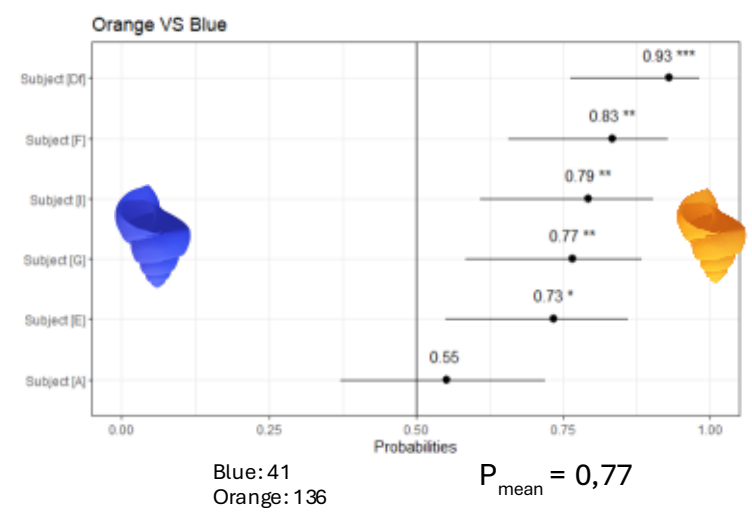

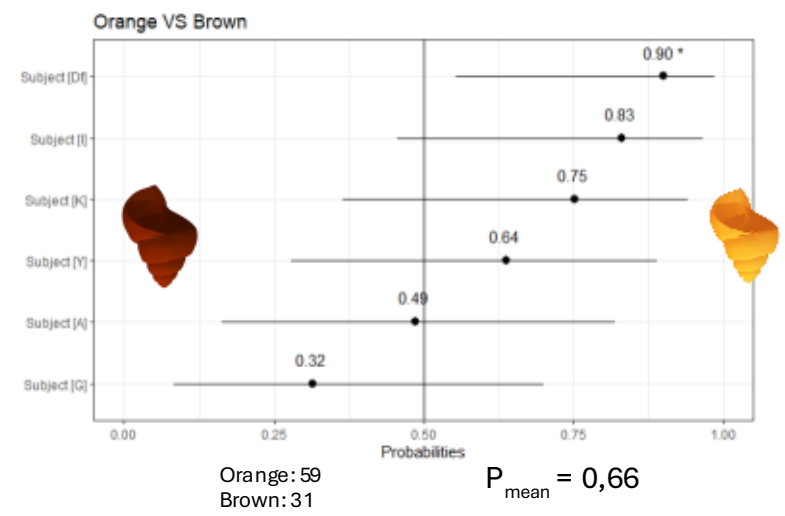

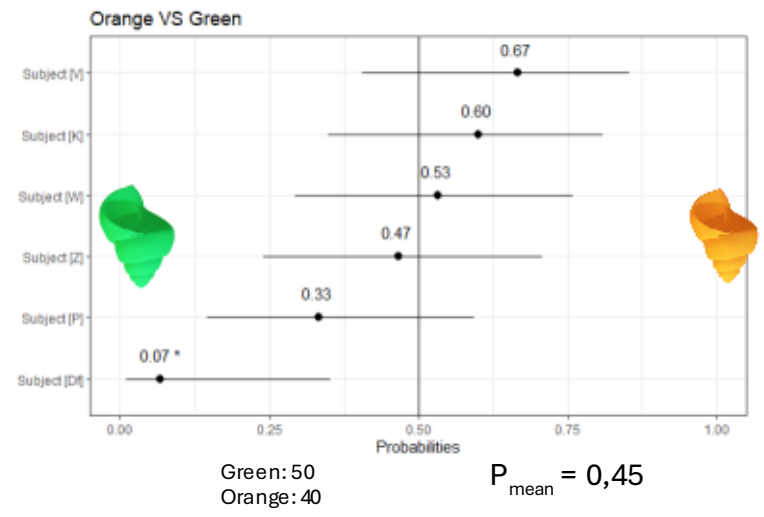

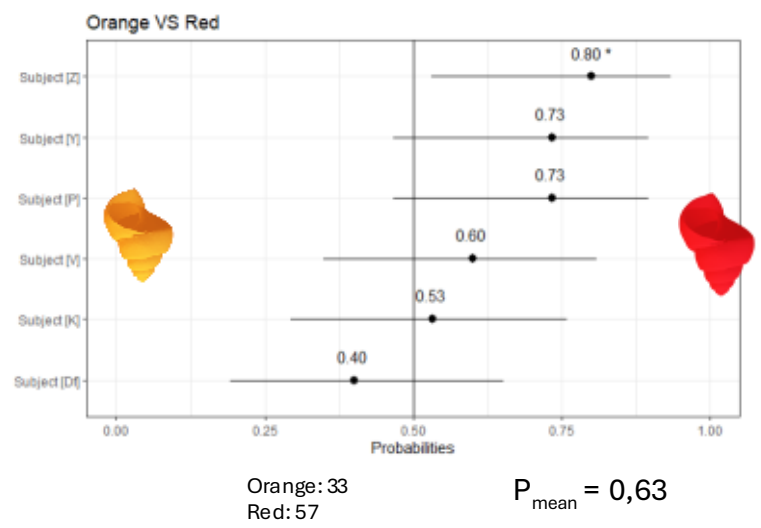

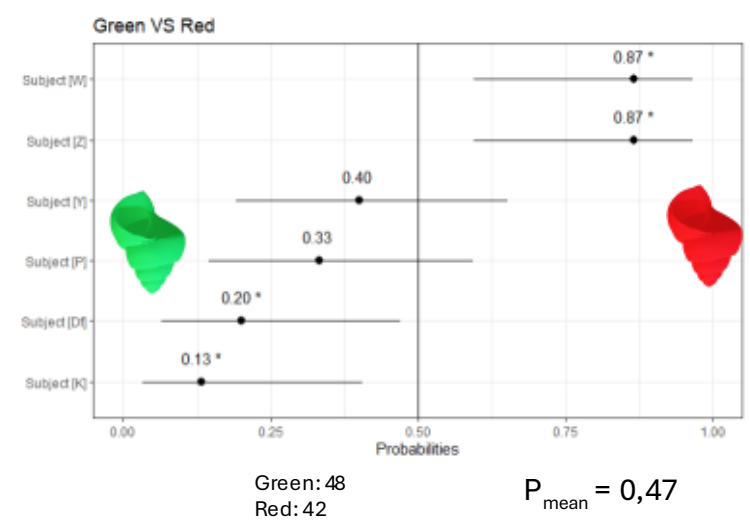

Size

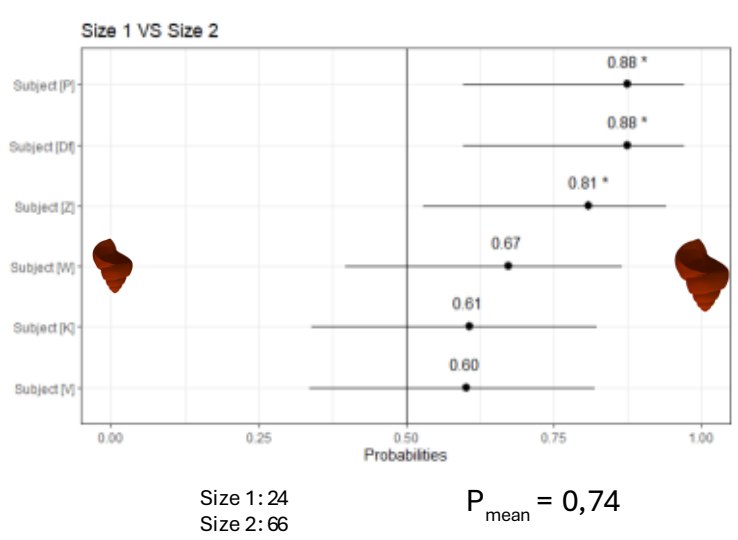

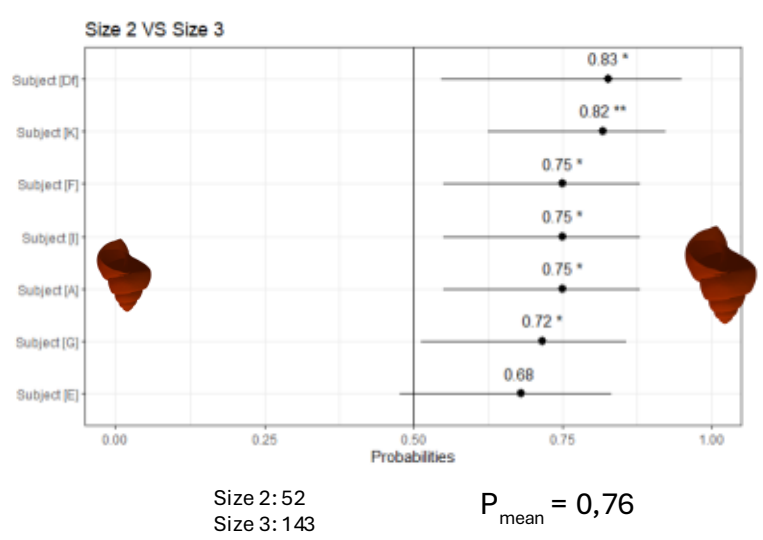

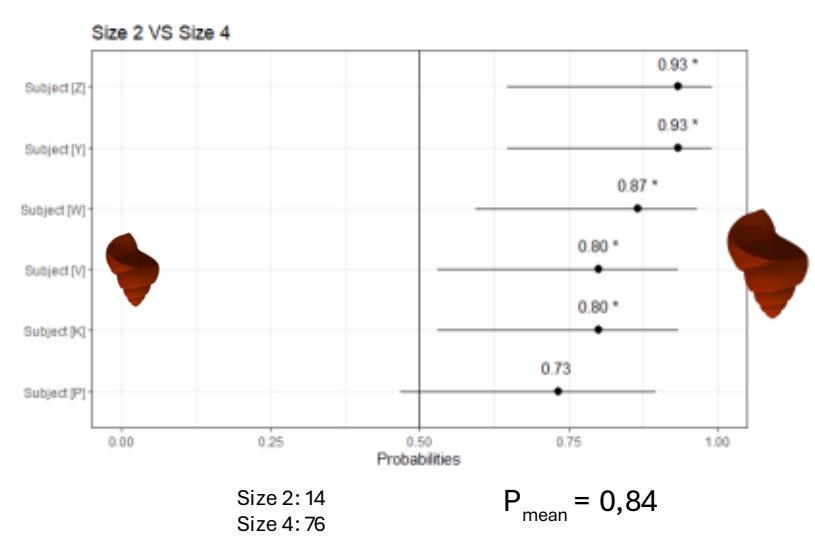

SECTION 3: Oddity effect

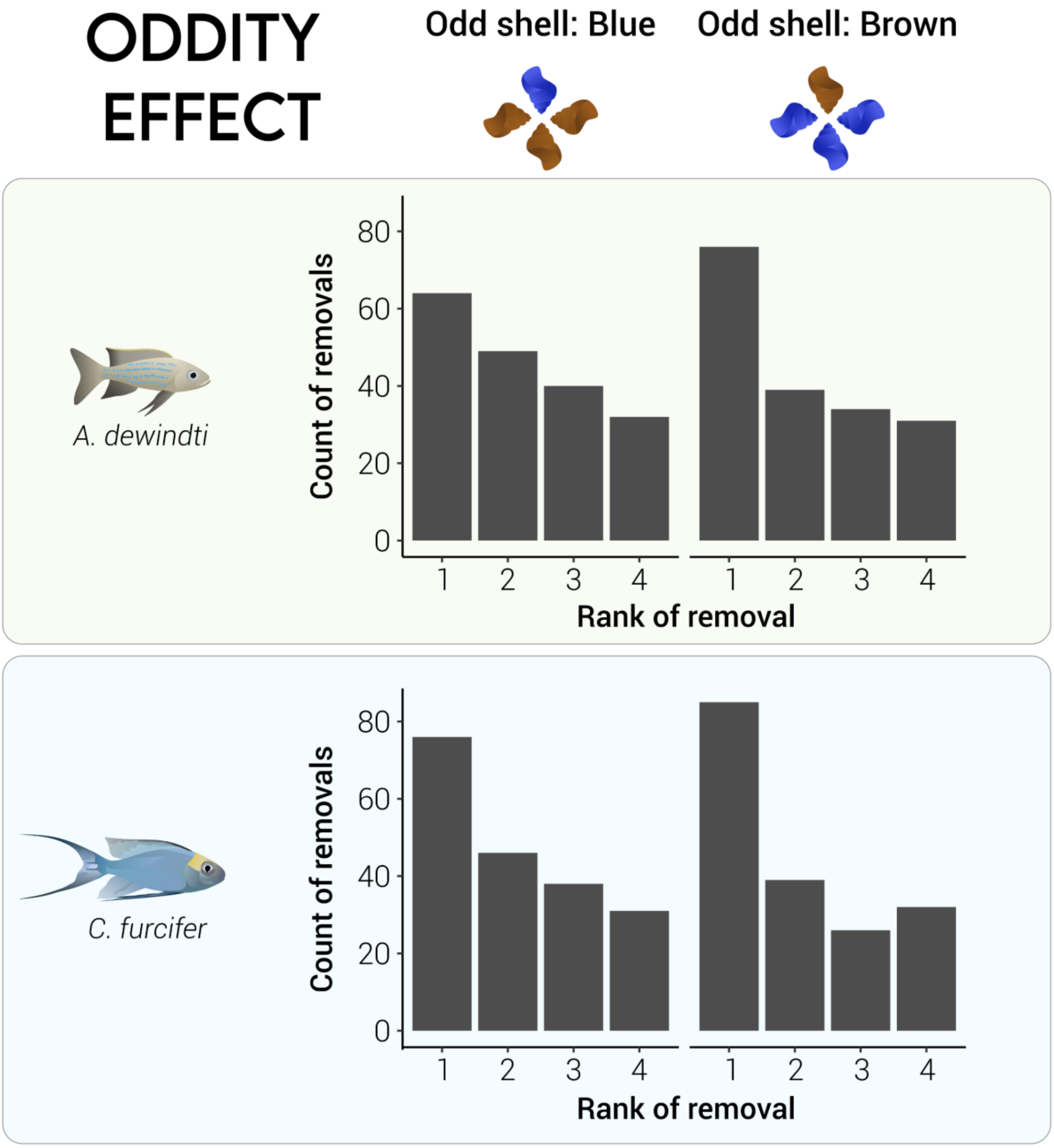

Figure S4: Both species show an oddity effect (low perceptual load situation). Counts of the different ranks of removal (odd shell removed first, second, third, or last) at the population-level. There is a significant effect of the rank in the whole population: both species significantly remove the odd shell first (GLMM Total of removals ~ Rank * Species * Colour of the odd shell + (1|Subject) with a Poisson distribution, (ANOVA $\chi^2 = 99.18$, $p<0.001$, n = 180 trials per species per odd shell colour).

Table S2: Results of the model investigating an oddity effect.

**Table S2** glmmTMB(Count of removals~ Rank * Species * Odd shell + (1|Subject), Poisson family)				
		Estimate ± SE	z-value	p-value
	Intercept	1.67 ± 0.13	13,39	**<0.001 *** **
**Rank**	Rank 2	-0.27 ± 0.19	-1,47	0.16
	Rank 3	-0.47 ± 0.20	-2,33	**<0.05 ***
	Rank	-0,69 ± 0.22	-3.20	**<0.01 ****
**Species**	C.furcifer	0.17 ± 0.17	1.01	0.31
**Odd shell**	Brown	0.17 ± 0.17	1.01	0.31
**Rank*Species**	Rank2*Furcif	-0.24 ± 0.27	-0.88	0.38
	Rank3*Furcif	-0.22 ± 0.28	-0.79	0.43
	Rank4*Furcif	-0.20 ± 0.30	-0.67	0.50
**Rank*Odd shell**	Rank2*Brown	-0.40 ± 0.27	-1.46	0.14
	Rank2*Brown	-0.33 ± 0.29	-1.16	0.25
	Rank4*Brown	-0.20 ± 0.30	-0.67	0.50
**Species*Odd.sh**	Furcif*Brown	-0.05 ± 0.23	-0.26	0.80
**Rank*Sp*Od.sh**	Rank2*Furcif*Br	0.12 ± 0.38	0.32	0.75
	Rank3*Furcif*Br	-0.16 ± 0.42	-0.38	0.71
	Rank4*Furcif*Br	0.12 ± 0.43	0.29	0.77
**Random effect**				
	Variance	Sd		
**Subject**	<0,001	<0,001		
**Type II ANOVA**				
		Df	Chi square	Pr(>Chisq)
Rank		3	99.18	**<0.001 *** **
Species		1	0.09	0.77
Odd shell		1	0.03	0.61
Rank * Species		3	2.19	0.53
Rank * Odd shell		3	5.23	0.16
Species * Odd shell		1	0.07	0.80
Rank * Species * Odd shell		3	0.47	0.93

Figure S5: Results of the oddity effect tests at the individual level.

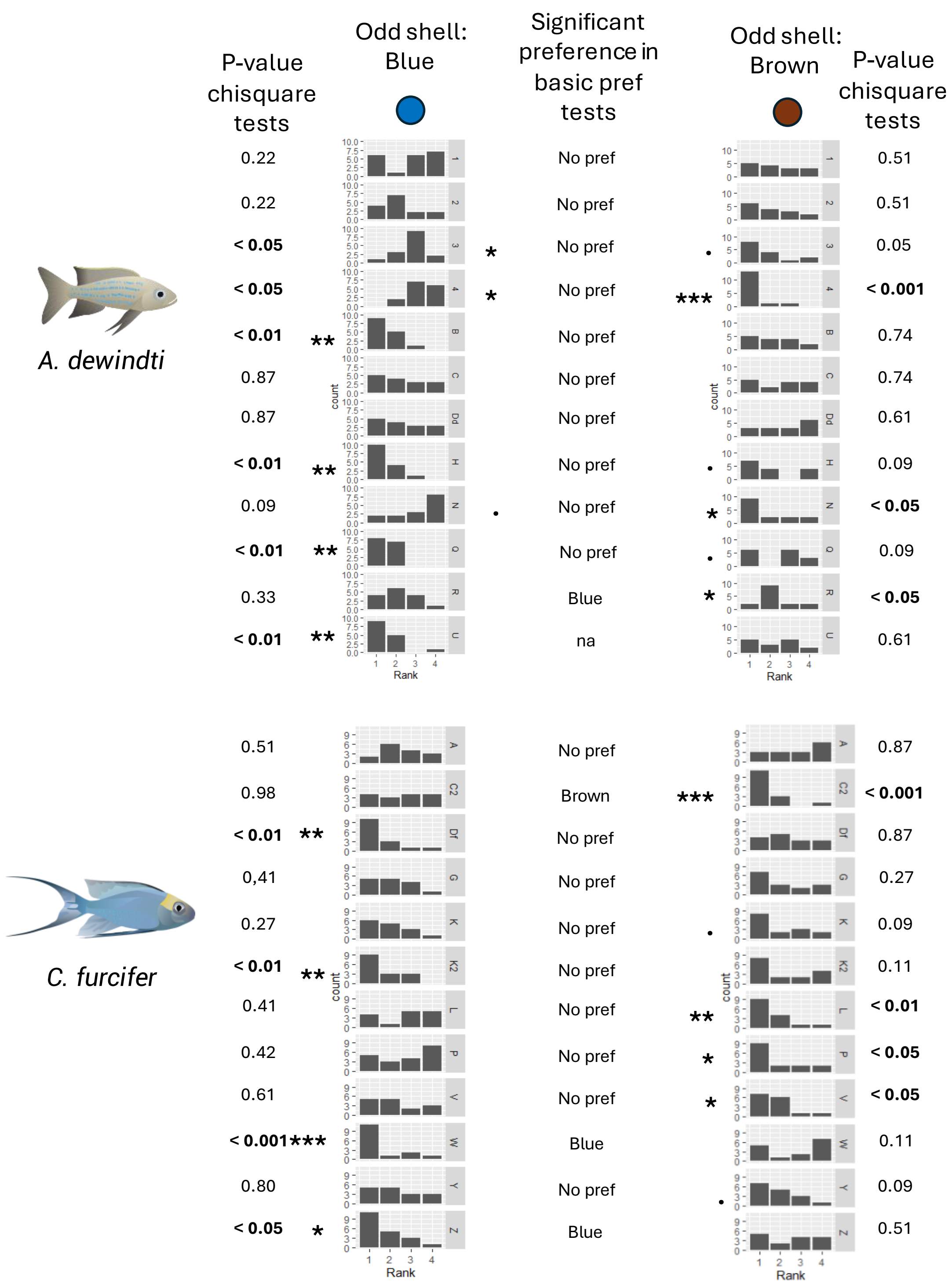

SECTION 4: Feature conjunctions

Table S3. Results of the multi-attribute binary choices. See table S1 for details. $^{+}$Model without the *C. furcifer* individual outlier presenting a significant preference for the red 2cm shell.

**FEATURE CONJUNCTION (HIGH PERCEPTUAL LOAD)** (binary choices)										
**Colour VS Size** (Shells)										
Option A	Option B	Species	Number of choices		Number of individuals			Interspecific difference		
			A	B	Preference for A	No preference	Preference for B	Estimate	Standard error	p-value
Brown 2.5cm	Orange 2cm	Dewindti	156	164	1	13	2	1.54	0.46	**<0.001** ***
		Furcifer	33	127	0	2	6			
Brown 3cm	Orange 2cm	Furcifer	73	87	0	5	3	-	-	-
Green 2cm	Orange 3cm	Dewindti	114	196	0	13	3	-0.69<br>-1.06^{+}	0.40<br>0.34^{+}	0.09<br>**<0.01**$^{+}$ **
Red 2cm	Orange 3cm	Furcifer	39	121	1	1	6			

Figure S6: Results of the binary choices in feature conjunction binary choices for *A. dewindti* and *C. furcifer*: Models: Choice ~ Subject + (1|Session/Trial), Binomial family

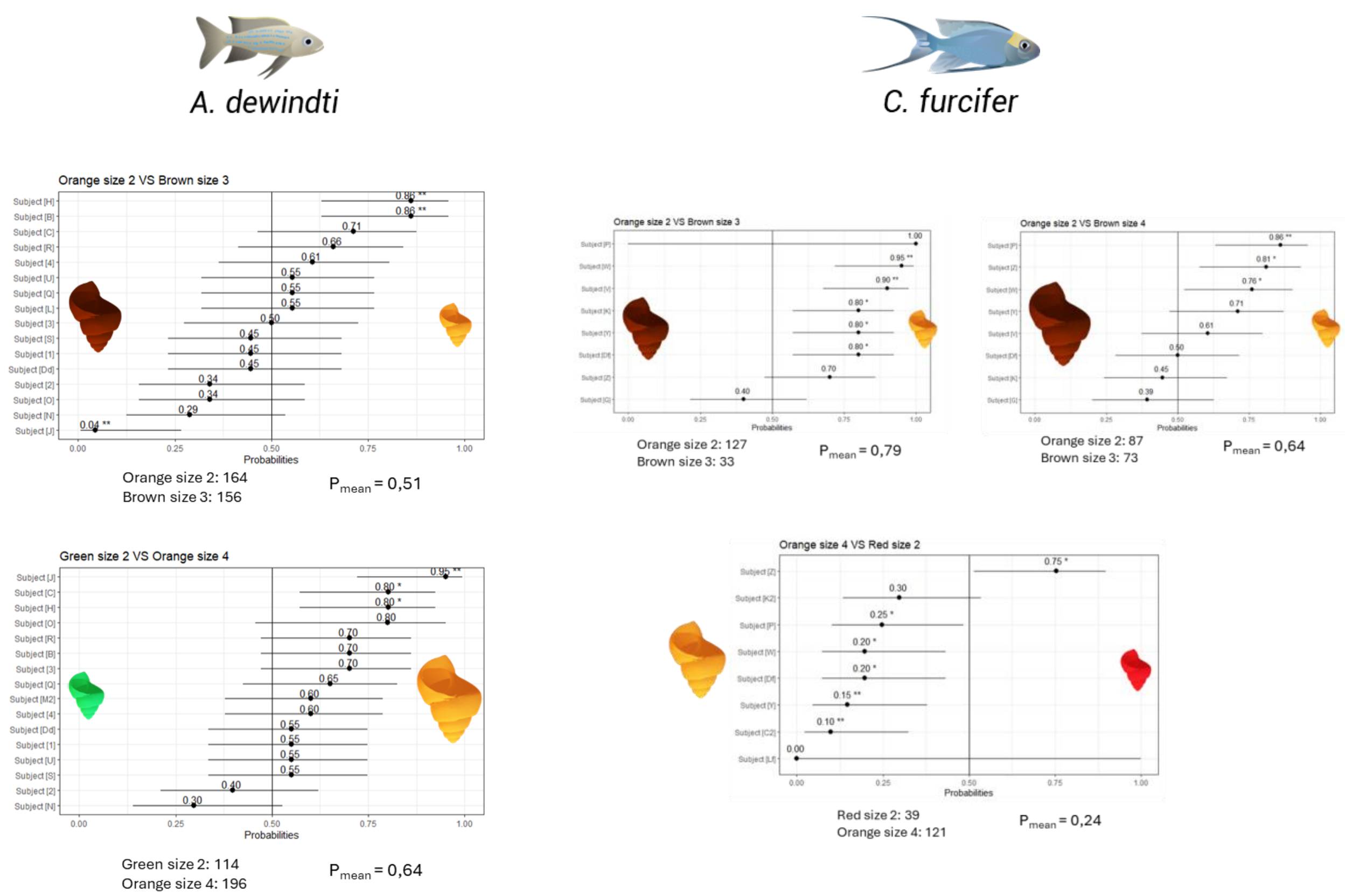

Table S4: Post-hoc analyses of the cox regression model for removal time of objects.

**Table S4** lsmeans(pairwise ~ Perceptual load | Species) after a cox regression model coxme(Removal time ~ Perceptual load*Species + (1|Subject) Contrasts High perceptual load – Low perceptual load (log scale)			
Species	Estimate ± SE	z-ratio	p-value
A. dewindti	-0.50 ± 0.08	-6.23	**<0.001 *****
C. furcifer	0.43 ± 0.09	4.92	**<0.001 *****

Table S5: Post-hoc analyses of the generalized linear model for manipulations of objects.

**Table S3B** lsmeans(pairwise ~ Perceptual load | Species) after a GLMM (Number of manipulations ~ Perceptual load*Species + (1|Subject), negative binomial family) Contrasts High perceptual load – Low perceptual load (log scale)			
Species	Estimate ± SE	z-ratio	p-value
A. dewindti	0.21 ± 0.20	1.07	0.28
C. furcifer	-0.33 ± 0.20	-1.65	0.10 ·

SECTION 5: Decoy effect

Table S6: Results of the models for the decoy effect tests for *A. dewindti* in A. the size decoy test and B. the color decoy test

**Table S6A. Size decoy.** glmmTMB(Choice ~ Experimental condition * Subject – 1 + (1|Session/Trial), binomial family)				
		Estimate ± SE	z-value	p-value
**Experimental condition**	Baseline pref	-0.18 ± 0.48	-0.38	0.71
	Decoy test	0.90 ± 0.52	1.73	0.09
**Random effect**				
	Variance	Sd		
**Session**	0.02	0.14		
**Trial*Session**	0.09	0.30		
**Type II ANOVA**				
		Df	Chi square	Pr(>Chisq)
Experimental condition		2	0.90	0.64
Subject		13	26.94	**< 0.05 ***
Experimental condition * Subject		13	29.02	**< 0.01 ****
**Post-hoc tests – least-square means (Experimental condition | Subject)**				
Subject		Estimate ± SE	z-value	p-value
1		-1.07 ± 0.67	-1.60	0.11
2		-1.50 ± 0.69	-2.19	**< 0.05 ***
3		1.13 ± 0.69	1.63	0.11
4		0.83 ± 0.65	1.27	0.20
C		0.24 ± 0.69	0.35	0.73
Dd		0.00 ± 0.65	0.00	1.0
J		-2.36 ± 1.13	-2.08	**< 0.05 ***
L		-0.92 ± 0.69	-1.33	0.18
N		0.26 ± 0.72	0.36	0.72
O		-0.41 ± 0.65	-0.64	0.52
Q		0.21 ± 0.64	0.32	0.75
R		2.05 ± 0.74	2.79	**< 0.01 ****
S		1.21 ± 0.73	0.73	0.09
U		0.43 ± 0.65	0.67	0.50

**Table S6B. Color decoy.** glmmTMB(Choice ~ Experimental condition * Subject – 1 + (1|Session/Trial), binomial family)				
		Estimate ± SE	z-value	p-value
**Experimental condition**	Baseline pref	0.20 ± 0.45	0.44	0.66
	Decoy test	-0.29 ± 0.44	-0.56	0.51
**Random effect**				
	Variance	Sd		
**Session**	< 0.001	< 0.001		
**Trial*Session**	0.01	0.11		
**Type II ANOVA**				
		Df	Chi square	Pr(>Chisq)
Experimental condition		2	2.11	0.35
Subject		11	19.22	0.06
Experimental condition * Subject		11	9.55	0.57
**Post-hoc tests – least-square means (Experimental condition | Subject)**				
Subject		Estimate ± SE	z-value	p-value
1		0.49 ± 0.63	0.78	0.44
2		-0.81 ± 0.65	-1.26	0.21
3		0.44 ± 0.67	0.66	0.51
4		0.61 ± 0.64	0.95	0.34
B		0.65 ± 0.67	0.98	0.33
Dd		0.20 ± 0.64	1.39	0.16
M2		0.89 ± 0.64	1.39	0.16
N		0.00 ± 1.69	0.00	1.0
Q		1.03 ± 0.66	1.57	0.12
R		-1.35 ± 0.89	-1.52	0.13
S		0.41 ± 0.64	0.64	0.52
U		0.00 ± 0.64	0.00	1.0

SECTION 6: Investigations of shape preferences

In addition to colour and size preferences, we also investigated shape preferences. We used 3D-printed brown objects of various shapes (*Neothauma* shell, octahedron, dodecahedron, and sphere) and tested them in binary choices in the two species.

Table S7. Results of the basic preferences for shapes. Are indicated the number of total first removals in the whole population for each binary choice, the number of individuals presenting a significant preference for removing one option first or no preference (GLMM Object removed first ~ Subject + (1|Session/Trial), binomial distribution), and the results of the interspecific differences in decision patterns (GLMM Object removed first ~ Species + (1|Subject) + (1|Session/Trial), binomial distribution).

BASIC PREFERENCES (binary choices)										
**Shape** (Brown object, 2cm, 3cm for Dodecahedron - Sphere)										
Shape A	Shape B	Species	Number of choices		Number of individuals			Interspecific difference		
			A	B	Preference for A	No preference	Preference for B	Estimate	Standard error	p-value
Dodeca-hedron	Shell	Dewindti	59	31	0	5	1	-0.53	0.35	0.13
		Furcifer	57	48	0	5	1			
Octa-hedron	Shell	Dewindti	68	37	0	6	1	-0.09	0.31	0.78
		Furcifer	57	34	0	6	0			
Dodeca-hedron	Octa-hedron	Dewindti	49	41	0	6	0	0.19	0.31	0.54
		Furcifer	45	45	0	6	0			
Octa-hedron	Sphere	Dewindti	54	36	1	5	0	0.04	0.43	0.93
		Furcifer	52	38	1	6	0			

Figure S7: Results of the binary choices in basic shape preferences binary choices for *A. dewindti*: Models: Choice ~ Subject + (1|Session/Trial), Binomial family

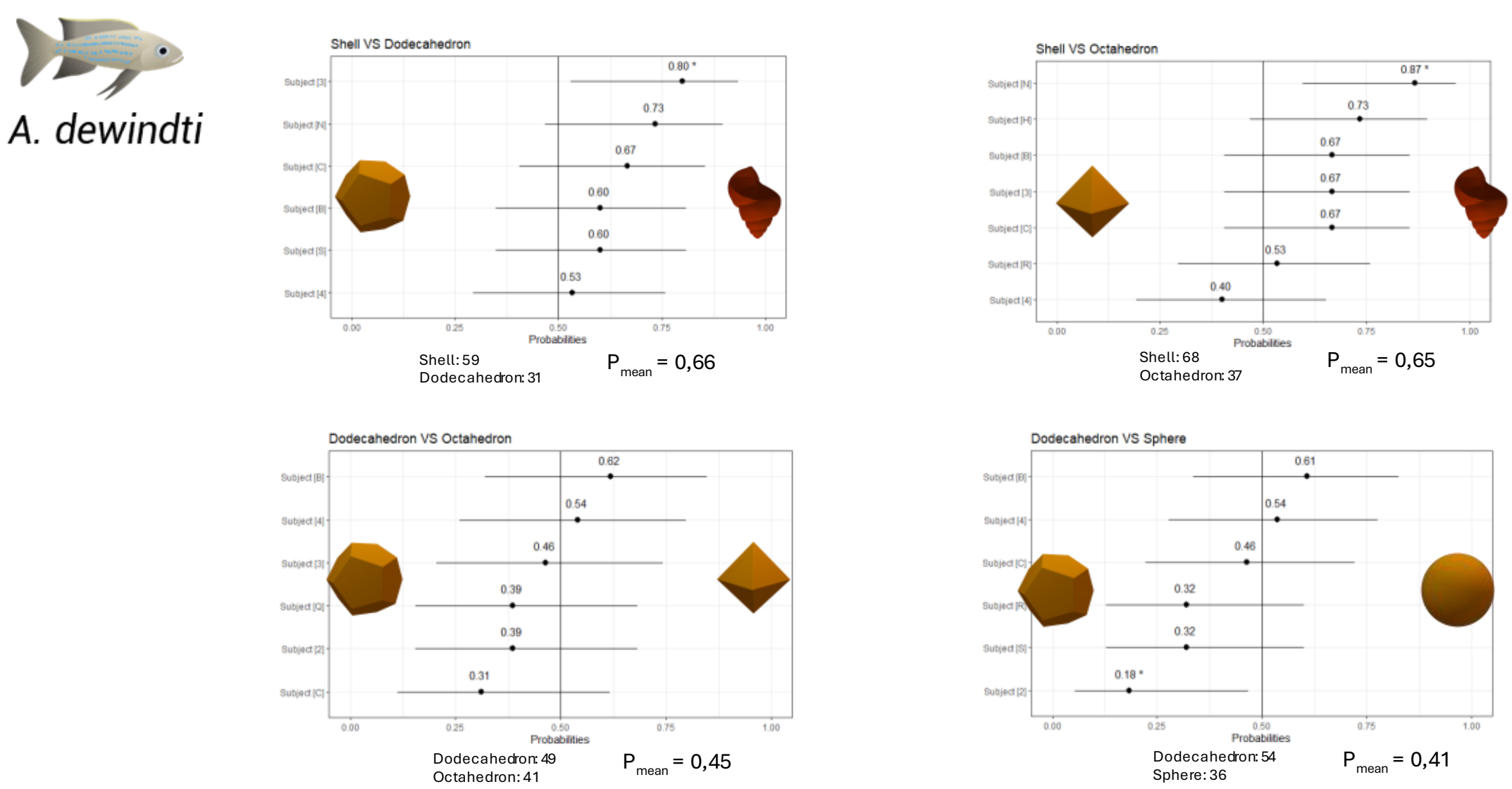

Figure S8: Results of the binary choices in basic shape preferences binary choices for *C. furcifer*: Models: Choice ~ Subject + (1|Session/Trial), Binomial family

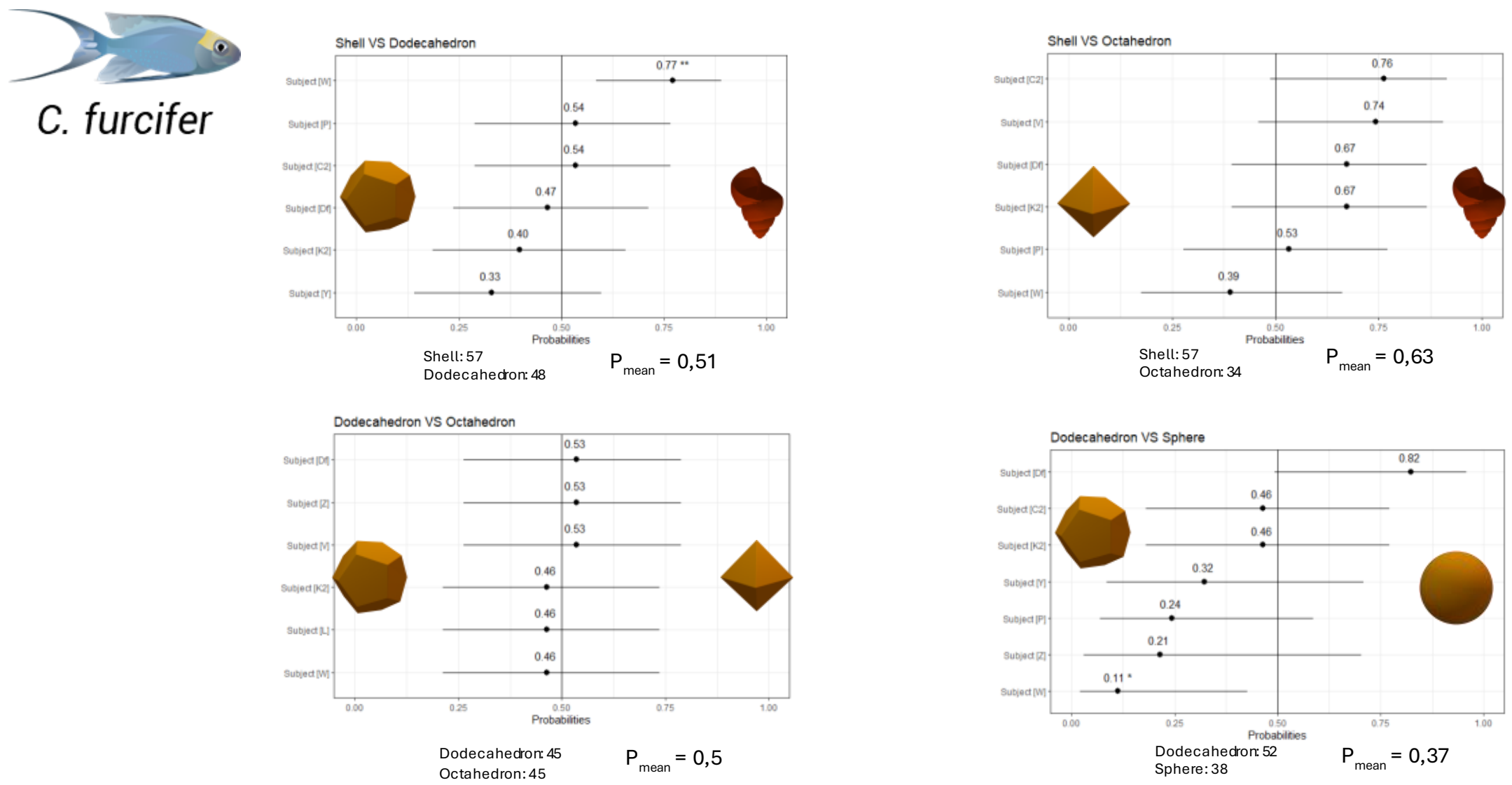

The two species presented similar preferences in shape. Preferences are rather weak. There is a preference for removing the shell first over a dodecahedron in *C. furcifer*. There is a similar preference in *A. dewindti*, as well as a preference to remove the shell first over an octahedron. Both species show a weak preference to remove an octahedron first over a sphere.
These results are consistent with previous observations that the two species preferred removing a shell over a stone when both objects were put in their bower (Tomasek et al., 2023; Tomasek et al., 2024).

Table S8. Results of the multi-attribute binary choices with shape and size. See table S7

**FEATURE CONJUNCTION (HIGH PERCEPTUAL LOAD)** (binary choices)										
**Shape VS Size** (Brown objects)										
Option A	Option B	Species	Number of choices		Number of individuals			Interspecific difference		
			A	B	Preference for A	No preference	Preference for B	Estimate	Standard error	p-value
Dodecah. 2cm	Shell 1.5cm	Dewindti	55	36	1	5	0	-0.30	0.39	0.44
		Furcifer	61	29	2	4	0			
Octahed. 2cm	Shell 1.5cm	Dewindti	57	33	1	5	0	0.00	0.31	1.0
		Furcifer	57	33	1	5	0			

Figure S9: Results of the binary choices in basic shape preferences binary choices for *A. dewindti*: Models: Choice ~ Subject + (1|Session/Trial), Binomial family

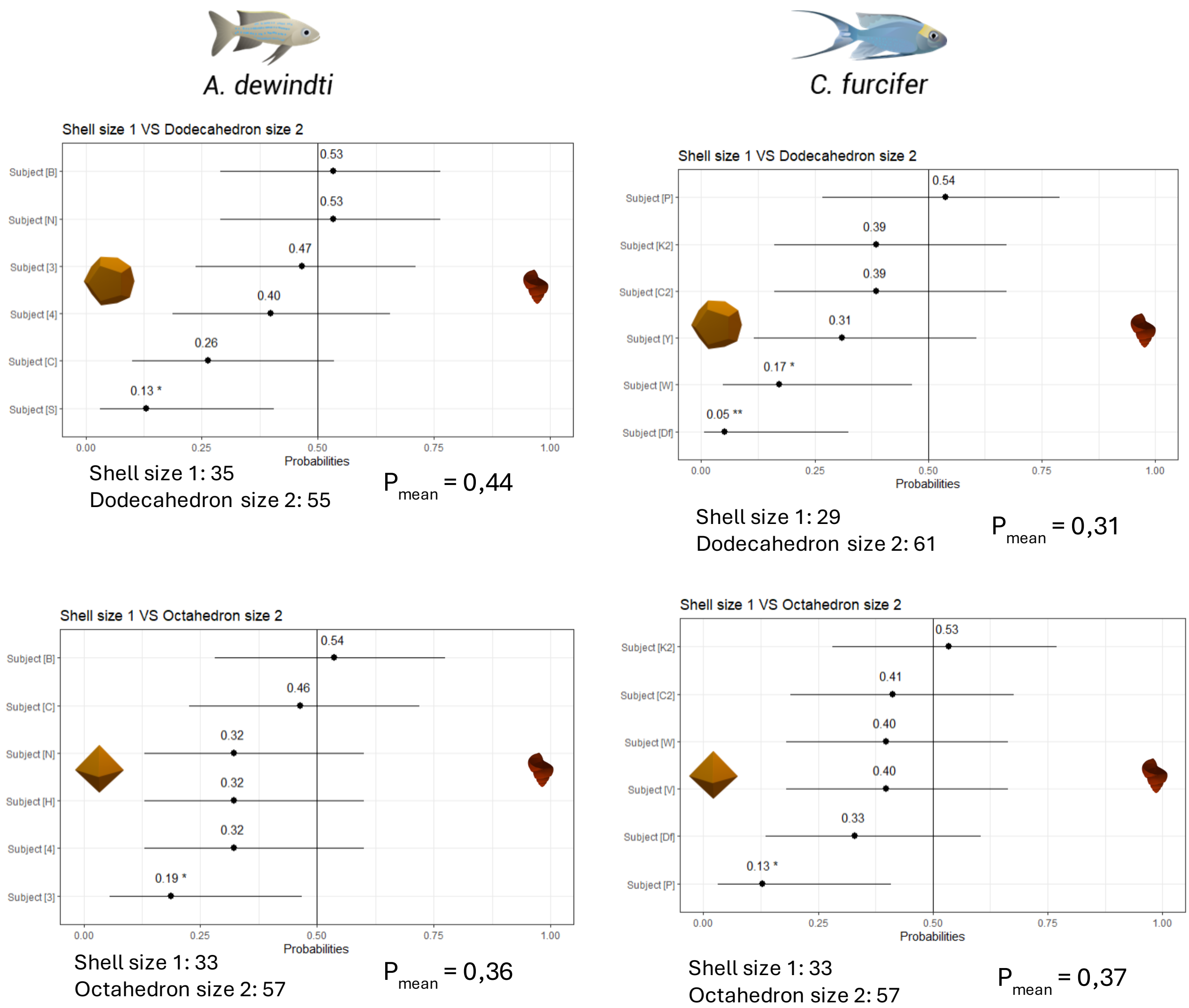

When the options conflicted in size and shape, some individuals of the two species showed a significant preference to remove a 2cm brown dodecahedron or a 2cm brown octahedron first (preferred size but unpreferred shapes) over a 1.5cm brown shell (preferred shape but unpreferred size). One individual out of six preferred to remove the dodecahedron or the octahedron first in all situations except for *C. furcifer* in which two individuals out of six significantly preferred to remove the dodecahedron first. Species did not differ significantly in their decisions patterns (generalised linear mixed-effect models, estimates = -0.30 ± 0.39 for dodecahedron and shell and 0.00 ± 0.31 for octahedron and shell, p-values = 0.44 and 1.0 respectively). In this situation, both species seem to use a similar strategy by prioritizing the size over the shape of objects.

Supplementary video: Videos of the series of experiments (binary choice task in low perceptual load, test of oddity effect, binary choice task in high perceptual load, test of decoy effect)

References:

Tomasek, M., Soller, K., Dufour, V., & Jordan, A. (2024). Differences in inhibitory control in two species of Tanganyikan bower-building cichlids contrasting in building flexibility. *Ecology and Evolution*, *14*(6), e11406. https://doi.org/10.1002/ECE3.11406

Tomasek, M., Stark, M., Dufour, V., & Jordan, A. (2023). Cognitive flexibility in a Tanganyikan bower-building cichlid, Aulonocranus dewindti. *Animal Cognition*, *1*, 1–13. https://doi.org/10.1007/S10071-023-01830-W
